# A novel lncRNA MCM3AP-AS1 promotes the growth of hepatocellular carcinoma by targeting miR-194-5p/FOXA1 axis

**DOI:** 10.1186/s12943-019-0957-7

**Published:** 2019-02-19

**Authors:** Yufeng Wang, Liu Yang, Tianxiang Chen, Xin Liu, Yang Guo, Qiaojuan Zhu, Xiangmin Tong, Wei Yang, Qiuran Xu, Dongsheng Huang, Kangsheng Tu

**Affiliations:** 1 Department of Hepatobiliary Surgery, the First Affiliated Hospital of Xi’an Jiaotong University, 277 Yanta West Road, Xi’an, 710061 Shaanxi Province China; 2 Key Laboratory of Tumor Molecular Diagnosis and Individualized Medicine of Zhejiang Province, Zhejiang Provincial People’s Hospital (People’s Hospital of Hangzhou Medical College), 158 Shangtang Road, Hangzhou, 310014 Zhejiang Province China; 3 Department of Neurosurgery, Zhejiang Provincial People’s Hospital (People’s Hospital of Hangzhou Medical College), 158 Shangtang Road, Hangzhou, 310014 Zhejiang Province China; 4 BengBu Medical College, Bengbu, 233030 Anhui Province China; 5 Department of Second Clinical Medical College, Zhejiang Chinese Medical University, Hangzhou, 310000 Zhejiang Province China

**Keywords:** Long non-coding RNA, Hepatocellular carcinoma, Tumorigenesis, miR-194-5p, FOXA1

## Abstract

**Background:**

Hepatocellular carcinoma (HCC) is the most common malignant liver tumor with poor clinical outcomes. Increasing amount of long non-coding RNAs (lncRNAs) have been revealed to be implicated in the carcinogenesis and progression of HCC. However, the expressions, clinical significances, and roles of most lncRNAs in HCC are still unknown.

**Methods:**

The expression of lncRNA MCM3AP antisense RNA 1 (MCM3AP-AS1) in HCC tissues and cell lines was detected by qRT-PCR and fluorescence in situ hybridization. Immunoblotting, CCK-8, EdU, colony formation and flow cytometry were performed to investigate the role of MCM3AP-AS1 in HCC cell proliferation, cell cycle and apoptosis in vitro. A subcutaneous tumor mouse model was constructed to analyze in vivo growth of HCC cells after MCM3AP-AS1 knockdown. The interactions among MCM3AP-AS1, miR-194-5p and FOXA1 were measured by RNA pull-down, RNA immunoprecipitation and luciferase reporter assay.

**Results:**

We revealed a novel oncogenic lncRNA MCM3AP-AS1, which is overexpressed in HCC and positively correlated with large tumor size, high tumor grade, advanced tumor stage and poor prognosis of HCC patients. MCM3AP-AS1 knockdown suppressed HCC cell proliferation, colony formation and cell cycle progression, and induced apoptosis in vitro, and depletion of MCM3AP-AS1 inhibited tumor growth of HCC in vivo. Mechanistically, MCM3AP-AS1 directly bound to miR-194-5p and acted as competing endogenous RNA (ceRNA), and subsequently facilitated miR-194-5p’s target gene forkhead box A1 (FOXA1) expression in HCC cells. Interestingly, FOXA1 restoration rescued MCM3AP-AS1 knockdown induced proliferation inhibition, G1 arrest and apoptosis of HCC cells.

**Conclusions:**

Our results recognized MCM3AP-AS1 as a novel oncogenic lncRNA, which indicated poor clinical outcomes in patients with HCC. MCM3AP-AS1 exerted an oncogenic role in HCC via targeting miR-194-5p and subsequently promoted FOXA1 expression. Our findings suggested that MCM3AP-AS1 could be a potential prognostic biomarker and therapeutic target for HCC.

**Electronic supplementary material:**

The online version of this article (10.1186/s12943-019-0957-7) contains supplementary material, which is available to authorized users.

## Background

According to the 2018 Global Cancer Statistics, about 841,000 newly diagnosed liver cancer cases and 782,000 liver cancer deaths were appeared worldwide, with China alone accounting for about 50% of the total number of cases and deaths [1, 2]. Hepatocellular carcinoma (HCC) accounts for 75–80% of all liver cancer cases [1]. Although a significantly decreasing incidence and mortality trend for HCC was observed in China, a huge population base and rapid population growth still led to a large and rising number of new HCC cases [3]. Thus, it is worth to better understand the molecular mechanisms underlying HCC tumorigenesis and progression, and develop more efficient targeted therapies for HCC.

Large amount of studies report that dysregulation of oncogenes and tumor suppressor genes contribute to HCC tumorigenesis and progression, but most of them focus on protein-coding genes [4]. Only 2% of the human genome accounts for protein coding genes, while about 70% of the genome is identified as non-coding RNAs (ncRNAs) due to the great progressions of genome and transcriptome sequencing [5]. ncRNAs are further grouped into long ncRNAs (lncRNA) and small ncRNAs depending on their transcript size [6]. LncRNAs, defined as a form of ncRNAs greater than 200 nt in length, are found to exert their gene transcription regulatory function by epigenetic regulatory mechanism [7]. Increasing evidences indicate that lncRNAs are implicated in several pathophysiological processes including human cancers [8–12]. Aberrant expression of lncRNAs has been frequently observed in cancers [13–16]. Moreover, lncRNAs regulate malignant behaviors of cancer cells, such as proliferation, apoptosis resistance, migration, invasion and drug resistance [17–20]. For instance, lncRNA miR503HG expression is found to be down-regulated in HCC and represses HCC metastasis via regulating the heterogeneous nuclear ribonucleoprotein A2/B1 (HNRNPA2B1)/nuclear factor κB (NF-κB) signaling [21]. High expression of lncRNA linc00210 is detected in liver cancer and contributes to tumor progression by driving the activation of Wnt/β-catenin pathway in a catenin beta interacting protein 1 (CTNNBIP1)-dependent manner [22]. Forkhead box A2 (FOXA2)-induced lncRNA-NEF is frequently down-regulated in HCC, and suppresses epithelial-mesenchymal transition (EMT) and tumor metastasis by antagonizing Wnt/β-catenin pathway [23]. Moreover, lncRNA-MUF is found to be highly expressed in HCC and facilitates hepatocarcinogenesis via directly regulating Annexin A2 (ANXA2)/Wnt/β-catenin signaling and miR-34a/Snail1/EMT axis [24]. In our previous study, we find that lncRNA TUSC7 is down-regulated in HCC and indicates poor prognosis of patients, and it inhibits EMT and HCC metastasis by acting as miR-10a sponge and subsequently leads to Eph tyrosine kinase receptor A4 (EphA4) upregulation [25]. Furthermore, we investigate the expression and function of lncRNA CASC2 in HCC and reveal that CASC2 exerts an anti-metastatic role by targeting miR-367/F-box and WD repeat domain containing 7 (FBXW7) axis [26]. Although several lncRNAs have been reported to participate in the tumorigenesis and progression of HCC, the expressions and roles of most lncRNAs in HCC are still unclear.

In this study, we analyzed differentially expressed lncRNAs in HCC compared to normal liver tissues based on the microarray data from National Center for Biotechnology Information (NCBI) Gene Expression Omnibus (GEO) dataset (GSE65485) and identified a novel highly expressed lncRNA MCM3AP antisense RNA 1 (MCM3AP-AS1) in HCC. Next, we investigated the expression, clinical significance, functional role and underlying mechanisms of MCM3AP-AS1 in HCC.

## Methods

### Clinical specimens

A total of 80 pairs of HCC and tumor-adjacent tissues were collected from patients who underwent hepatectomy at the First Affiliated Hospital of Xi’an Jiaotong University (Xi’an, China). None of HCC patients received any pre-operative treatments, such as radiofrequency ablation (RFA), transcatheter arterial chemoembolization (TACE), immunotherapy and targeted therapy. The tissue samples were confirmed by two histopathologists. All samples were immediately snap-frozen in liquid nitrogen and subsequently stored at − 80 °C until RNA extraction and protein isolation. The demographic and clinicopathological features for HCC patients were described in Table 1.

**Table 1:** Correlation between the clinicopathologic characteristics and MCM3AP-AS1 expression in hepatocellular carcinoma

Characteristics		*n* = 80	MCM3AP-AS1		*P*
			Low expression (*n* = 40)	High expression (*n* = 40)	
Age (y)	< 50	33	14	19	0.256
	≥50	47	26	21	
Sex	Male	65	31	34	0.390
	Female	15	9	6	
HBV	Absent	25	15	10	0.228
	Present	55	25	30	
Serum AFP level (ng/mL)	< 20	24	14	10	0.329
	≥20	56	26	30	
Tumor size (cm)	< 5	30	21	9	0.006^*^
	≥5	50	19	31	
No. of tumor nodules	1	66	34	32	0.556
	≥2	14	6	8	
Cirrhosis	Absent	33	19	14	0.256
	Present	47	21	26	
Venous infiltration	Absent	49	27	22	0.251
	Present	31	13	18	
Edmondson-Steiner grading	I + II	60	34	26	0.039^*^
	III + IV	20	6	14	
TNM tumor stage	I + II	61	36	25	0.004^*^
	III + IV	19	4	15	

*HBV* hepatitis B virus, *AFP* alpha-fetoprotein, *TNM* tumor-node-metastasis

^*^Statistically significant

### Cell culture

The human immortalized normal hepatocyte cell line LO2 and HCC cell lines HepG2, Hep3B, Huh7, SMMC-7721 were maintained in our lab [26]. The cells were cultured in Dulbecco’s Modified Eagle’s Medium (DMEM, Gibco BRL, Grand Island, NY, USA) supplemented with 10% fetal bovine serum (Gibco) and antibiotics (100 μg/mL streptomycin and 100 U/mL penicillin, Sigma, St-Louis, MO, USA) in a humidified incubator containing 5% CO_2_ at 37 °C.

### Plasmids and transfection

Two lentivector-mediated short-hairpin MCM3AP-AS1 (sh-MCM3AP-AS1–1 and sh-MCM3AP-AS1–2) and non-targeting plasmids (sh-control) were designed and synthesized by Geneseed Biotech (Guangzhou, China). Lentivirus infection of HCC cells was performed in the presence of Polybrene (8 ng/ml). The cDNA encoding MCM3AP-AS1 was PCR-amplified by the Thermo Scientific Phusion Flash High-Fidelity PCR Master Mix (Thermo-Fisher Scientific, Waltham, MA, USA) and subcloned into the pcDNA3.1 plasmid (Invitrogen, Carlsbad, CA, USA). The empty plasmid pcDNA3.1 was used as negative control (EV). Hsa-miR-194-5p mimics and negative control mimics were obtained from Guangzhou RiboBio Co., Ltd. (Guangzhou, China). The plasmid expressing FOXA1 was previously described [27]. A small interfering RNA (siRNA) targeting AGO2 and scrambled siRNA were obtained from Geneseed Biotech. Plasmids were transfected into cells using Lipofectamine 2000 (Invitrogen) following the manufacturer’s protocol.

### Quantitative real-time polymerase chain reaction (qRT-PCR)

TRIzol reagent (Invitrogen) was used for total RNA isolation from HCC tissues and cultured cells. Total RNA was reverse transcribed into cDNA using a RevertAid First Strand cDNA Synthesis Kit (Thermo-Fisher Scientific). qRT-PCR analyses were performed using SYBR® Premix Ex Taq™ II (Takara, Dalian, China) and Taqman UniversalMaster Mix II (Life Technologies Corporation, Carlsbad, CA, United States) on an ABI PRISM 7300 Sequence Detection system (Applied Biosystems, Foster City, CA, USA) in accordance with the manufacturers’ instructions. The 2^-ΔΔCt^ method was used to calculate the relative gene expression normalized by GAPDH and U6. The sequences of the primers were listed in Table 2.

**Table 2:** Primers for qRT-PCR

Gene name		Primer sequences (5′ to 3′)
MCM3AP-AS1	Forward	GCTGCTAATGGCAACACTGA
	Reverse	AGGTGCTGTCTGGTGGAGAT
miR-194-5p	Forward	CTAGTACCTAGAGGAACCTTTGAAGACTGTTACAGCTCAGCA
	Reverse	AGCTTGCTGAGCTGTAACAGTCTTCAAAGGTTCCTCTAGGTA
miR-23c	Forward	CCAGAAGGACGTAGAAG
	Reverse	CTTCACTGTGATGGGCTC
TRIP12	Forward	CCGGGGCCCAACCACAAGAC
	Reverse	TGGACGCTGAACGGGAACGC
CUL4B	Forward	TGCTGCTCAGGAGGTCAGATC
	Reverse	TGGAATCAAAGTCTTCTCTCTCGTT
FOXA1	Forward	GCAATACTCGCCTTACGGCT
	Reverse	TACACACCTTGGTAGTACGCC
BTBD7	Forward	AGTCAAATGCCTGGTTACGG
	Reverse	TGTCTGGCACATTGGACATT
HBEGF	Forward	CCATTCTGAAAGGCTGGTTTG
	Reverse	TACTCCGGAAGGGTCCTTTGT
U6	Forward	GTGGACCGCACAAGCTCGCT
	Reverse	TTGTTGAACGGCACTGTGTATAGCA
GAPDH	Forward	CAGGAGGCATTGCTGATGAT
	Reverse	GAAGGCTGGGGCTCATTT

### Western blot analysis

HCC tissues and cells were lysed with RIPA buffer (Beyotime, Shanghai, China) and protein concentrations were quantified with a BCA protein assay kit II (BIO-RAD, Hercules, CA, USA). Protein samples were separated by 10% SDS-PAGE gel and transferred onto a nitrocellulose membrane (Invitrogen). The membranes were incubated with rabbit-anti-human FOXA1 (ab170933; Abcam, Cambridge, MA, USA), rabbit-anti-human PARP1 (ab191217; Abcam), rabbit-anti-human caspase-3 (ab32351; Abcam), rabbit-anti-human caspase-7 (ab32522; Abcam), rabbit-anti-human Cyclin D1 (ab134175; Abcam), rabbit-anti-human p21 (ab109520; Abcam), mouse-anti-human β-actin (ab8226; Abcam) and mouse-anti-human GAPDH primary antibody (sc-47,724; Santa Cruz Biotechnology, Santa Cruz, Dallas, TX, USA) overnight at 4 °C. Horseradish peroxidase (HRP)-conjugated secondary antibodies (NXA931–1ML and NA934-1ML, GE Healthcare Life Sciences, Beijing, China) were used to incubate the membranes for 1 h at room temperature. The blot signals on the membrane were visualized with ECL reagents (Millipore, Plano, TX, USA) and detected using Amersham™ Imager 680 from GE Healthcare Life Sciences.

### Cell counting Kit-8 (CCK-8) assay

HCC cells suspensions were added to a 96-well plate at a density of 1 × 10^4^/mL. A total of 10 μL of CCK-8 solution (Dojindo, Tokyo, Honshu, Japan) was added to each well at the same time every day for 3 days. Finally, the absorbance at 450 nm was measured using a microplate reader (Thermo-Fisher Scientific) after a 2 h incubation.

### Colony formation assay

Forty-eight hours after transfection, HCC cells (1 × 10^3^ per well) were seeded in a 6-well plate and cultured with complete medium for 2 weeks. Cell colonies were fixed with 4% paraformaldehyde for 30 min and stained with 0.5% crystal violet for 30 min at room temperature.

### Ethynyl deoxyuridine (EdU) incorporation assay

EdU incorporation assay was performed with the EdU kit (Roche, Indianapolis, IN, USA) in accordance with the manufacturer’s instruction. Results were acquired using the Zeiss fluorescence photomicroscope (Carl Zeiss, Oberkochen, Germany) and quantified via counting at least five random fields.

### Flow cytometry assay

For cell cycle analysis, cells were collected and fixed using 70% ethanol. After washing with PBS and subsequently washing with stain buffer, 1 × 10^6^ cells were resuspended in 0.5 mL of PI/RNase Staining Buffer (BD biosciences, San Jose, CA, USA), and cells were incubated for 15 min at room temperature (RT), protected from light. A FACSCanto II flow cytometer (BD biosciences) was used to analyze cell cycle distribution. For apoptosis assay, the PE Annexin V Apoptosis Detection Kit I (BD biosciences) was used following the manufacturer’s protocols. Cells were harvested and washed with pre-cold PBS buffer twice. Then, 5 μl of PE Annexin V and 5 μl of 7-AAD solution were added to each sample, and cells were incubated for 15 min at RT. FACSCanto II flow cytometer was used to measure the cell apoptosis.

### Tumor xenograft model

BALB/c nude mice (male, 4–5-week-old, 18-20 g) were obtained from Shanghai SLAC Laboratory Animal Co. Ltd. (Shanghai, China) and randomly divided into two groups (*n* = 6 per group). Hep3B cells (1 × 10^6^ per injection) that were transfected with sh-MCM3AP-AS1 and sh-control, respectively, were implanted into the right flank of the mice via subcutaneous injection. Tumor volumes were measured every 3 days after being apparently observed and calculated with the following formula: Volume = (length × width^2^)/2. After 3 weeks, all mice were sacrificed under anesthesia. Tumor tissues were harvested and subjected to immunohistochemistry for Ki-67 staining [28]. The animal experiments were approved by the Animal Care and Use Committee of Xi’an Jiaotong University.

### Pull-down assay with biotinylated miR-194-5p

HepG2 cells were transfected with biotinylated wild type (wt) miR-194-5p, mutant (mt) miR-194-5p and negative control (NC) (Guangzhou RiboBio Co., Ltd). Cell lysates were harvested 48 h after transfection and incubated with Dynabeads M-280 Streptavidin (Invitrogen, CA, USA) for 3 h at 4 °C according to the manufacturer’s protocol. Then, the beads were washed three times with ice-cold lysis buffer and once with high salt buffer (0.1% SDS, 1% Triton X-100, 2 mM EDTA, 20 mM Tris-HCl, pH 8.0 and 500 mM NaCl) [29]. The bound RNAs were purified using TRIzol for the qRT-PCR analysis.

### RNA immunoprecipitation (RIP)

RIP assay was performed with the EZ-Magna RIP Kit (Millipore, Bedford, MA, USA) and a AGO2 antibody (Millipore) as previously described [26]. qRT-PCR was carried out to detect co-precipitated RNAs.

### Luciferase reporter assay

The sequence of 3′-UTR of FOXA1 or MCM3AP-AS1 was amplified from human genomic DNA. Then these sequences were respectively subcloned into pGL3 luciferase reporter vector (Promega, Madison, WI, USA). The potential miR-194-5p binding sites were mutated by the Quick-change site-directed mutagenesis kit (Agilent Technologies, Santa Clara, CA, USA). The wt (mt) 3′-UTR of FOXA1 vector or wt (mt) MCM3AP-AS1 vector and control mimics or miR-194-5p mimics were co-transfected into HepG2 and SMMC-7721 cells. The luciferase activity was measured and normalized as previously described [26].

### RNA fluorescent in situ hybridization

Cy3-labeled MCM3AP-AS1 probe were obtained from RiboBio (Guangzhou, China). Subcellular localization of MCM3AP-AS1 was detected by the FISH Kit (RiboBio, Guangzhou, China) according to the manufacturer’s instructions [30].

### Quantitation of MCM3AP-AS1 and miR-194-5p expression levels

The exact copy numbers of MCM3AP-AS1 and miR-194-5p transcripts per Hep3B and HepG2 cell were quantified by using quantitative real-time RT-PCR assay. In this assay, serially diluted RT-PCR products of MCM3AP-AS1 and miR-194-5p were used as templates to formulate standard curves, and then, the exact copies of MCM3AP-AS1 and miR-194-5p per cell were calculated accordingly.

### Statistical analysis

Data were analyzed using GraphPad Prism 6.0 Software (GraphPad Inc., San Diego, CA, USA). The Student’s t-test was used to analyze differences between two groups, and two-way ANOVA was used when more than two groups were compared. The correlations between MCM3AP-AS1 and miR-194-5p expression were analyzed using the Pearson correlation test. Overall survival curves were protracted using the Kaplan-Meier method and estimated by the log-rank test. Differences were defined as statistically significant if *P* < 0.05.

## Results

### A novel lncRNA MCM3AP-AS1 is overexpressed in HCC

**Fig. 1:**
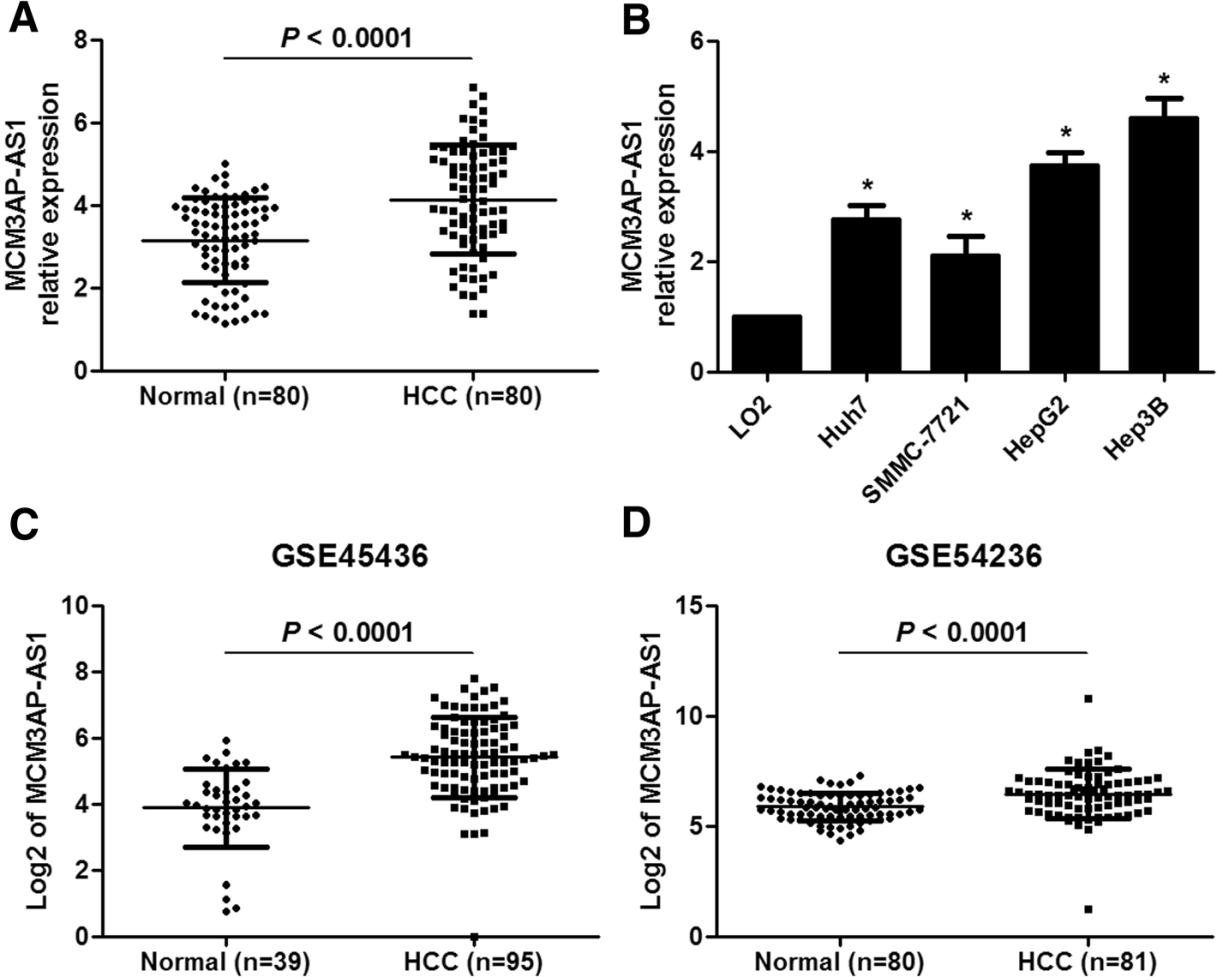
MCM3AP-AS1 expression is up-regulated in HCC. **a** The expression of MCM3AP-AS1 in 80 pairs of HCC and matched noncancerous tissues was measured by qRT-PCR. *P* < 0.0001 by Student’s t-test. **b** The expressions of MCM3AP-AS1 in human normal hepatocyte cell line LO2 and HCC cell lines Huh7, SMMC-7721, HepG2 and Hep3B were detected using qRT-PCR. *P < 0.05 by Student’s t-test versus LO2. **c** and **d** Two GEO datasets (GSE45436 and GSE54236) from R2: Genomics Analysis and Visualization Platform (http://r2.amc.nl) indicated that MCM3AP-AS1 expression was prominently higher in HCC tissues compared to normal liver tissues. *P* < 0.0001 by Student’s t-test

To further disclose differentially expressed lncRNAs in HCC, we analyzed a microarray data comparing the expression of lncRNAs in 50 HCC tissues and 5 adjacent non-tumor tissues from the GEO database with the accession number GSE65485. Thirty-seven lncRNAs were differentially expressed in HCC tissues compared to adjacent non-tumor tissues (Table 3). A novel lncRNA MCM3AP-AS1 (also known as MCM3APAS), which was 2.84-fold higher in HCC tissues than that in adjacent non-tumor tissues, caught our attention. Next, we search for the expression pattern of MCM3AP-AS1 in HCC based on TCGA data from starBase V3.0 [31]. The results showed that MCM3AP-AS1 in HCC tissues was significantly higher than that in normal liver tissues (*P* < 0.0001, Additional file 1: Figure S1). Furthermore, qRT-PCR analysis of MCM3AP-AS1 in 80 pairs of HCC and matched tumor-adjacent tissues revealed that MCM3AP-AS1 was significantly overexpressed in HCC tissues compared to tumor-adjacent tissues (P < 0.0001, Fig. 1a). Elevated expression of MCM3AP-AS1 also observed in HCC cell lines (HepG2, Hep3B, SMMC-7721 and Huh7) compared to LO2 cells (*P* < 0.05, Fig. 1b). Moreover, based on two other GEO datasets (GSE45436 and GSE54236) from R2: Genomics Analysis and Visualization Platform (http://r2.amc.nl), we found that MCM3AP-AS1 expression was prominently higher in HCC tissues compared to normal liver tissues (P < 0.0001, Fig. 1c and d). Thus, these results indicated that MCM3AP-AS1 upregulation was a frequent event in HCC.

**Table 3:** Differentially expressed lncRNAs between HCC tissues and adjacent non-tumor tissues in GSE65485 dataset

LncRNA	Fold change (tumor vs. normal)	*P* value	FDR
FAM99A	0.04	0.000	0.010
LOC646982	0.08	0.001	0.024
DIO3OS	0.16	0.005	0.061
PWRN1	0.31	0.010	0.096
LOC286002	0.43	0.005	0.061
NEAT1	0.45	0.009	0.094
LOC100009676	1.79	0.003	0.044
LOC440944	1.81	0.006	0.069
TUG1	1.83	0.005	0.061
LOC202781	1.92	0.001	0.024
HCG18	1.93	0.001	0.024
DGCR11	2.18	0.002	0.031
SNHG12	2.27	0.002	0.035
LOC728190	2.29	0.006	0.068
LOC100302401	2.33	0.006	0.068
SNHG10	2.33	0.003	0.044
LOC220930	2.34	0.008	0.088
LOC388796	2.47	0.000	0.010
LOC100134713	2.50	0.003	0.044
LOC100130581	2.55	0.001	0.024
LOC100128191	2.61	0.008	0.081
C6orf164	2.83	0.010	0.096
**MCM3AP-AS1**	**2.84**	**0.001**	**0.024**
SNHG1	2.87	0.000	0.007
SNHG3	3.08	0.000	0.015
LOC150381	3.08	0.005	0.061
LOC144486	4.35	0.000	0.010
LOC541471	5.17	0.001	0.024
LOC100133612	5.85	0.000	0.010
LOC92659	6.44	0.000	0.007
LOC84931	6.62	0.003	0.044
LOC284551	6.84	0.001	0.018
LOC150197	7.00	0.003	0.044
PVT1	7.07	0.000	0.018
CDKN2B-AS1	7.17	0.001	0.018
LOC286467	8.24	0.006	0.069
SNHG4	8.64	0.000	0.007

Bold indicates interested lncRNA

### High level of MCM3AP-AS1 correlates with poor prognosis of HCC patients

**Fig. 2:**
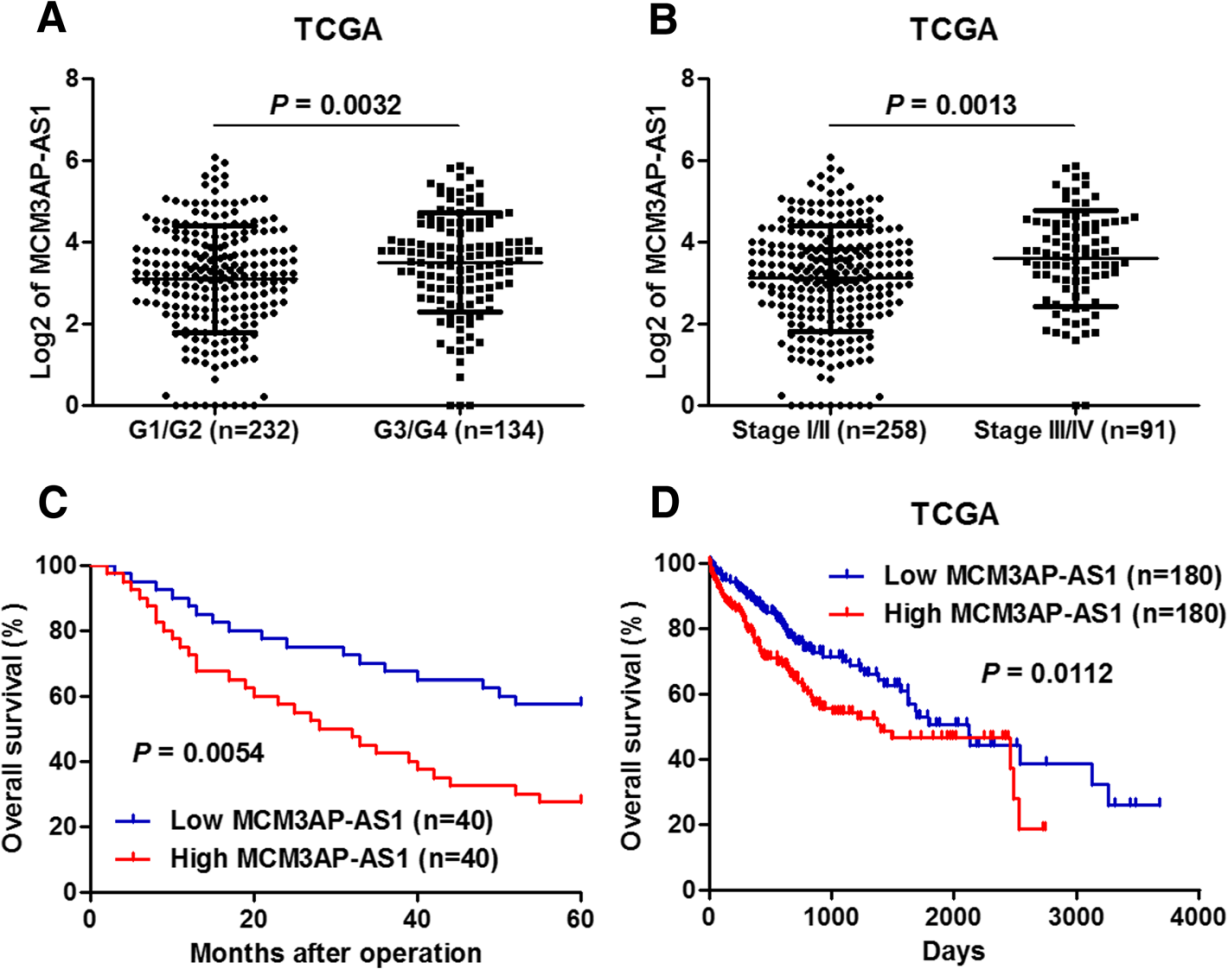
The clinical significance of MCM3AP-AS1 in HCC. **a** Based on TCGA data from R2: Genomics Analysis and Visualization Platform (http://r2.amc.nl), the expression of MCM3AP-AS1 in 232 cases of HCC with low tumor grades (G1-G2) and 134 samples of HCC with high tumor grades (G3-G4). *P* = 0.0032 by Student’s t-test. **b** The expression of MCM3AP-AS1 in 258 cases of HCC with early tumor stages (I-II) and 91samples of HCC with advanced tumor stages (III-IV). *P* = 0.0013 by Student’s t-test. **c** Kaplan-Meier survival analysis revealed that HCC patients with high MCM3AP-AS1 expression showed a significant poorer overall survival compared to those with low MCM3AP-AS1 expression. The median expression level of MCM3AP-AS1 was used as the cut-off. *P* = 0.0054 by Log-rank test. **d** TCGA data from OncoLnc (http://www.oncolnc.org/) further demonstrated that high MCM3AP-AS1 expression also indicated poor survival of HCC patients. The median expression level of MCM3AP-AS1 was used as the cut-off. *P* = 0.0112 by Log-rank test

Next, we aimed to reveal the clinical significance of MCM3AP-AS1 in HCC. TCGA data from R2: Genomics Analysis and Visualization Platform (http://r2.amc.nl) revealed that MCM3AP-AS1 was more highly expressed in HCC with high tumor grades (G3 + G4) than that in HCC with low tumor grades (G1 + G2) (*P* = 0.0032, Fig. 2a). Furthermore, MCM3AP-AS1 was also more highly expressed in HCC with advanced tumor stages (III-IV) than that in HCC with early tumor stages (I-II) (*P* = 0.0013, Fig. 2b). We divided HCC patients into tow subgroups (low/high MCM3AP-AS1 level) by using the median of the cohort as a cut-off value. As shown in Table 1, the correlation analysis between MCM3AP-AS1 expression and clinicopathologic characteristics of these 80 HCC patients indicated that high expression of MCM3AP-AS1 was positively correlated with large tumor size (*P* = 0.006), high tumor grade (*P* = 0.039), and advanced TNM stages (*P* = 0.004). Kaplan-Meier survival analysis showed that HCC patients with high MCM3AP-AS1 expression had a significant poorer overall survival than those with low MCM3AP-AS1 expression (*P* = 0.0054, Fig. 2c). Furthermore, TCGA data from OncoLnc (http://www.oncolnc.org/) further demonstrated that high MCM3AP-AS1 expression also indicated poor survival of HCC patients (*P* = 0.0112, Fig. 2d). Collectively, our data showed that high MCM3AP-AS1 expression was associated with poor clinical outcomes of HCC patients.

### Depletion of MCM3AP-AS1 suppresses cell growth and induces apoptosis of HCC cells

**Fig. 3:**
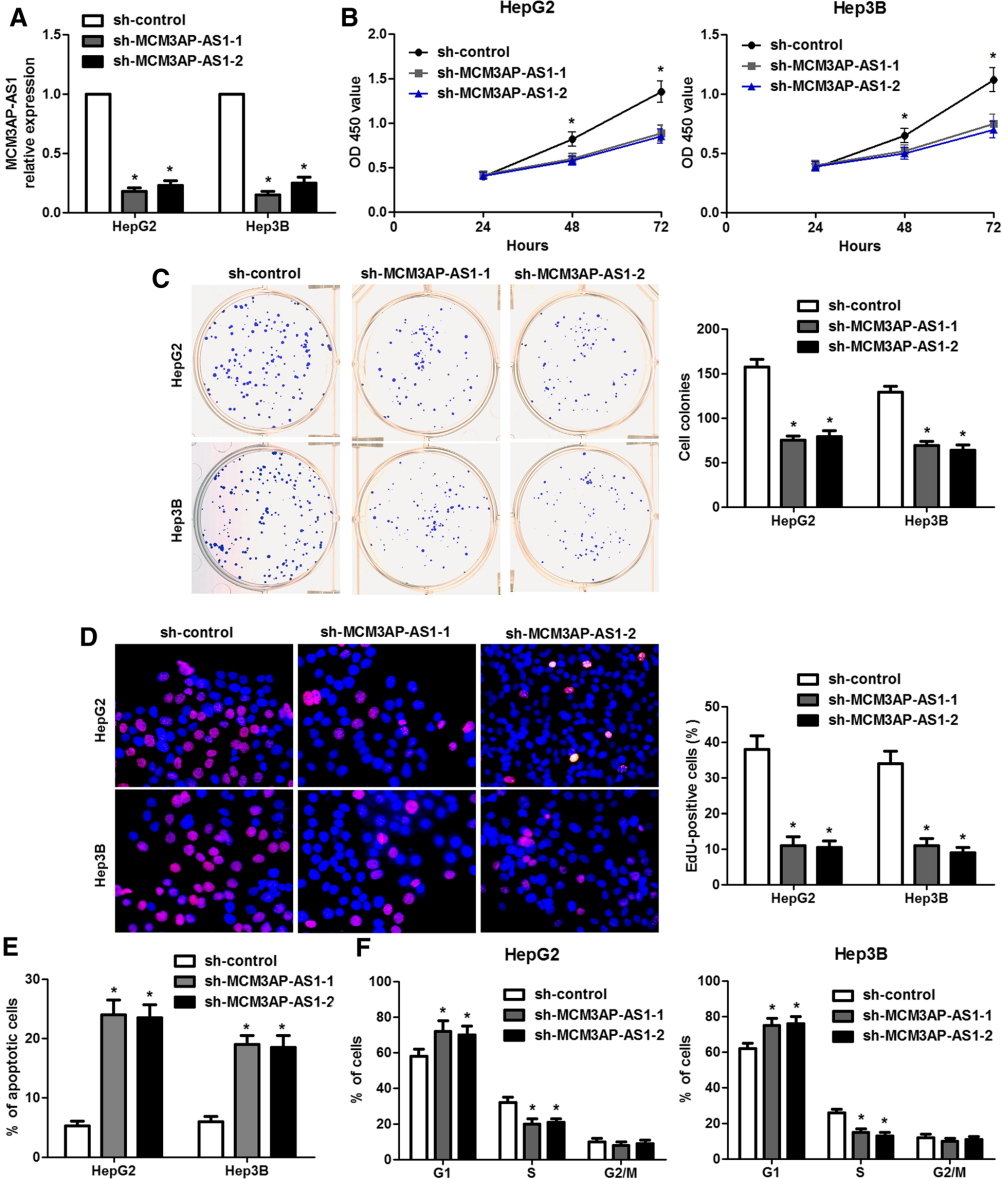
MCM3AP-AS1 knockdown suppresses HCC cell proliferation and cell cycle progression and induces apoptosis in vitro. **a** The expression of MCM3AP-AS1 was knocked down by two different shRNAs in both HepG2 and Hep3B cells. *P < 0.05 by Student’s t-test versus sh-control. **b** CCK-8 assay indicated that MCM3AP-AS1 knockdown repressed HCC cell proliferation. *P < 0.05 by ANOVA versus sh-control. **c** The number of HCC cell colonies was reduced after MCM3AP-AS1 knockdown. *P < 0.05 by Student’s t-test versus sh-control. **d** The proliferation of HCC cells with MCM3AP-AS1 knockdown was decreased compared to control cells as detected by EdU incorporation assay. *P < 0.05 by Student’s t-test versus sh-control. **e** Flow cytometry assay revealed that the percentage of apoptotic HCC cells was increased by MCM3AP-AS1 knockdown. *P < 0.05 by Student’s t-test versus sh-control. **f** MCM3AP-AS1 knockdown led to G1 arrest in both HepG2 and Hep3B cells. *P < 0.05 by Student’s t-test versus sh-control

Since MCM3AP-AS1 expression was associated with tumor size, we next disclosed the biological roles of MCM3AP-AS1 in HCC cell growth. MCM3AP-AS1 were stably depleted in HepG2 and Hep3B cells with different specific shRNAs (*P* < 0.05, Fig. 3a). CCK-8 assays indicated that MCM3AP-AS1 knockdown significantly inhibited the proliferation of HepG2 and Hep3B cells (P < 0.05, Fig. 3b). Colony formation and EdU incorporation assays also indicated that MCM3AP-AS1 silencing prominently suppressed the growth of HepG2 and Hep3B cells (*P* < 0.05. Figure 3c and d). Moreover, flow cytometry assays revealed that the percentage of apoptotic HCC cells were obviously increased by MCM3AP-AS1 knockdown (*P* < 0.05. Figure 3e). Depletion of MCM3AP-AS1 led to cell cycle arrest at G1 phase in HepG2 and Hep3B cells (*P* < 0.05, Fig. 3f). Furthermore, MCM3AP-AS1 knockdown led to increased levels of cleaved PARP1, cleaved caspase-3, cleaved caspase-7 and p21, and decreased Cyclin D1 expression in HCC cells (P < 0.05, Additional file 2: Figure S2). Notably, MCM3AP-AS1 knockdown did not significantly affected the growth of LO2 cells, which had low MCM3AP-AS1 expression (Additional file 3: Figure S3). Thus, these results showed that knockdown of MCM3AP-AS1 repressed the proliferation, cell cycle progression and induced apoptosis of HCC cells in vitro.

### MCM3AP-AS1 knockdown restrains tumorigenesis of HCC in vivo

**Fig. 4:**
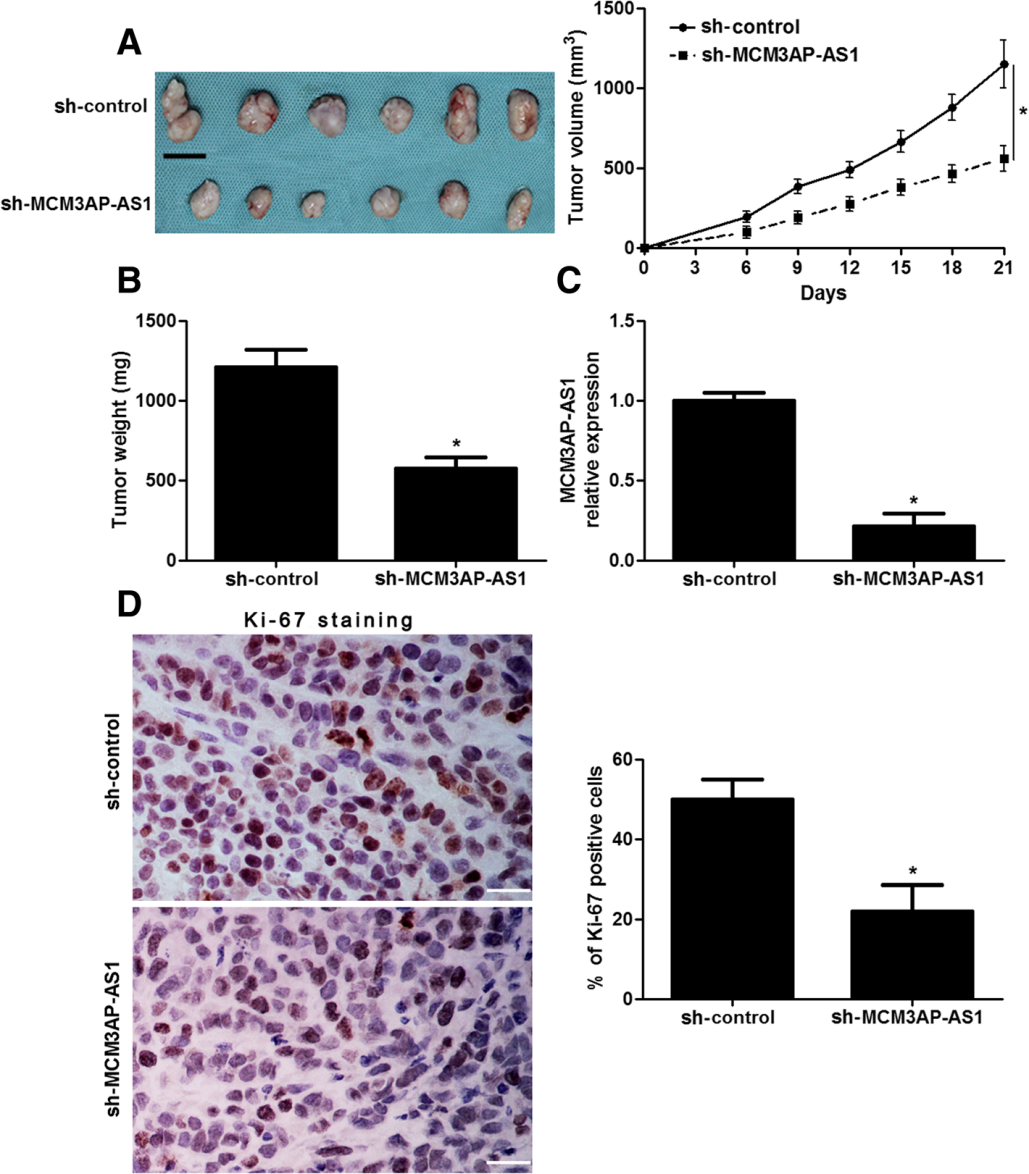
MCM3AP-AS1 knockdown suppresses HCC growth in vivo. **a** Hep3B cells that were stably transfected with MCM3AP-AS1 shRNA (*n* = 6) or control shRNA (*n* = 6) were subcutaneously injected into nude mice. Tumor growth curves indicated that MCM3AP-AS1 knockdown led HCC growth restriction in mice. *P < 0.05 by ANOVA. Scale bar: 1 cm. **b** The subcutaneous tumors were harvested and weighted at the 21st day after implantation. *P < 0.05 by Student’s t-test. **c** Xenograft tissues were subjected to qRT-PCR for MCM3AP-AS1 expression. *P < 0.05 by Student’s t-test. **d** Immunostaining of Ki-67 in xenograft tissues arising from MCM3AP-AS1 knockdown group and control group. Scale bar: 50 μm. *P < 0.05 by Student’s t-test

To further elucidate the biological roles of MCM3AP-AS1 in HCC tumorigenesis in vivo, Hep3B cells with MCM3AP-AS1 knockdown were implanted into nude mice via subcutaneous injection. The results of tumor growth curves and tumor weight indicated that MCM3AP-AS1 knockdown obviously reduced tumor growth in mice (*P* < 0.05, Fig. 4a and b). Tumor tissues were harvested for qRT-PCR analysis of MCM3AP-AS1. We confirmed that lower expression of MCM3AP-AS1 was detected in tumor tissues arising from MCM3AP-AS1 knockdown group compared to control group (*P* < 0.05, Fig. 4c). Ki-67 immunostaining indicated that the subcutaneous tumors formed by MCM3AP-AS1 knockdown Hep3B cells showed less Ki-67 positive cells compared to those formed by control Hep3B cells (P < 0.05, Fig. 4d). Altogether, these results suggested that MCM3AP-AS1 knockdown suppressed HCC tumorigenesis in vivo.

### MCM3AP-AS1 acts as a molecular sponge for miR-194-5p in HCC cells

**Fig. 5:**
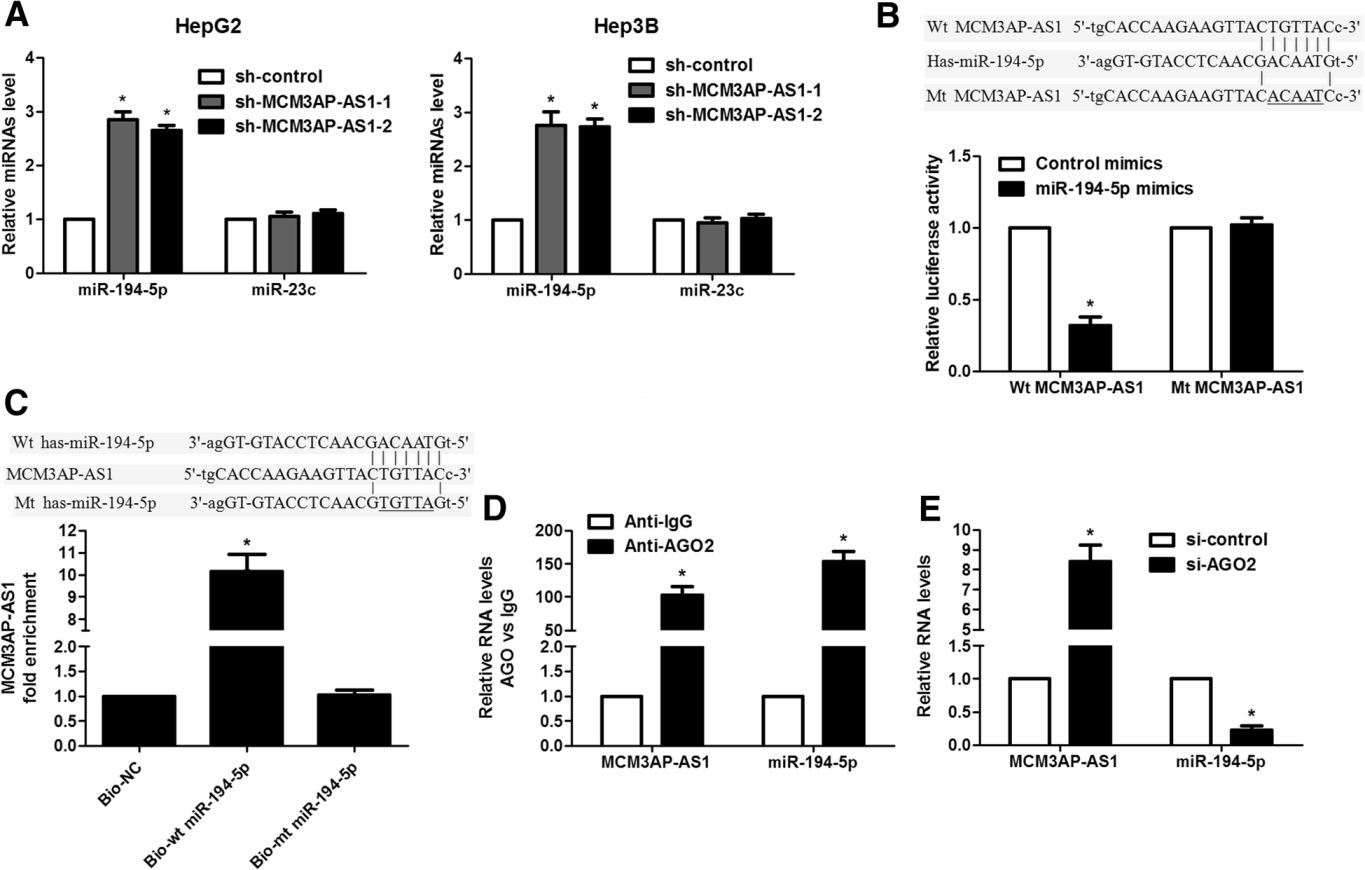
MCM3AP-AS1 functions as molecular sponge for miR-194-5p in HCC cells. **a** HepG2 and Hep3B cells that were transfected with two different MCM3AP-AS1 shRNAs and control shRNA, respectively, were subjected to qRT-PCR for miRNAs expression. *P < 0.05 by Student’s t-test versus sh-control. (**b**) Complementary sequence between miR-194-5p and wild type (wt) MCM3AP-AS1. The putative binding sites of miR-194-5p was mutated in MCM3AP-AS1 (mt MCM3AP-AS1). SMMC-7721 cells that were co-transfected with miR-194-5p mimics and wt or mt MCM3AP-AS1 vector were measured for luciferase activity. *P < 0.05 by Student’s t-test. **c** The sequences for wt and mt forms of miR-194-5p were shown. MCM3AP-AS1 was highly enriched in the sample pulled down by biotinylated wt miR-194-5p rather than mt miR-194-5p. *P < 0.05 by Student’s t-test versus Bio-NC. **d** Anti-AGO2 RIP was performed in HepG2 cells transiently overexpressing miR-194-5p. The results indicted that MCM3AP-AS1, miR-194-5p and AGO2 formed a complex in miR-194-5p-transfected HepG2 cells. *P < 0.05 by Student’s t-test. **e** HepG2 cells that were transfected with AGO2 siRNA or control siRNA were subjected to qRT-PCR for MCM3AP-AS1 and miR-194-5p expression. *P < 0.05 by Student’s t-test

To investigate the mechanisms underlying the role of MCM3AP-AS1 in HCC, we predicted 31 miRNAs containing binding site of MCM3AP-AS1 by using starBase V3.0. Among these miRNAs, we found that only low expression of miR-194-5p and miR-23c associated with poor prognosis of HCC patients based on TCGA data from OncoLnc (*P* < 0.05, Additional file 4: Figure S4). Thus, we detected expression of miR-194-5p and miR-23c in HepG2 cells after MCM3AP-AS1 knockdown. Our data revealed that MCM3AP-AS1 knockdown significantly increased miR-194-5p expression rather than miR-23c (*P* < 0.05, Fig. 5a). Consistent results were confirmed in Hep3B cells (P < 0.05, Fig. 5a). The expression of miR-194-5p in tumor tissues from MCM3AP-AS1 knockdown injected mice was significantly higher than that in control mice (*P* < 0.05, Additional file 5: Figure S5A). Reduced expression of miR-194-5p was confirmed in HCC tissues compared to paracancerous tissues (*P* < 0.0001, Additional file 6: Figure S6A). Analysis of TCGA data and our HCC cases indicated a significant inverse correlation between MCM3AP-AS1 and miR-194-5p expression (*P* < 0.05, Additional file 6: Figure S6B and S6C). Furthermore, luciferase reporter assay revealed that miR-194-5p overexpression reduced the luciferase activity of vectors containing wt MCM3AP-AS1, but it did not influence the luciferase activity of vectors containing mt MCM3AP-AS1 in both SMMC-7721 and HepG2 cells (P < 0.05, Fig. 5b and Additional file 7: Figure S7A). Notably, we found that MCM3AP-AS1 level was reduced after overexpression of miR-194-5p in HCC cells (*P* < 0.05, Additional file 7: Figure S7B). Moreover, MCM3AP-AS1 was pulled down by biotin-labeled miR-194-5p, while mutagenesis of the binding sites for MCMC3AP-AS1 in miR-194-5p disrupted the interaction between MCM3AP-AS1 and miR-194-5p (*P* < 0.05, Fig. 5c). MCM3AP-AS1 mainly located in the cytoplasm (Additional file 8: Figure S8A). qRT-PCR found that the expression level of MCM3AP-AS1 was approximately 100 copies per cell, and mature miR-194-5p levels were approximately 200 copies per cell (Additional file 8: Figure S8B), suggesting that the abundance of MCM3AP-AS1 and miR-194-5p was comparable. Thus, our data demonstrated that MCM3AP-AS1 was associated with miR-194-5p and acted as a competing endogenous RNA (ceRNA) in HCC cells. Since AGO2 is an critical component of the RNA-induced silencing complex (RISC) and acts as a key regulator of miRNA functions [32]. We conducted anti-AGO2 RIP in HepG2 cells transiently overexpressing miR-194-5p. Endogenous MCM3AP-AS1 pull-down by AGO2 was significantly enriched in miR-194-5p-transfected cells (*P* < 0.05, Fig. 5d). In addition, AGO2 silencing impaired AGO2 dependent degradation of MCM3AP-AS1 and resulted in increased expression of MCM3AP-AS1, whereas miR-194-5p stability was reduced by AGO2 knockdown in HepG2 cells (*P* < 0.05, Fig. 5e). These data demonstrated that miR-194-5p bound to MCM3AP-AS1 and induced the degradation of MCM3AP-AS1 in HCC cells.

### FOXA1 is a novel target of miR-194-5p in HCC cells

**Fig. 6:**
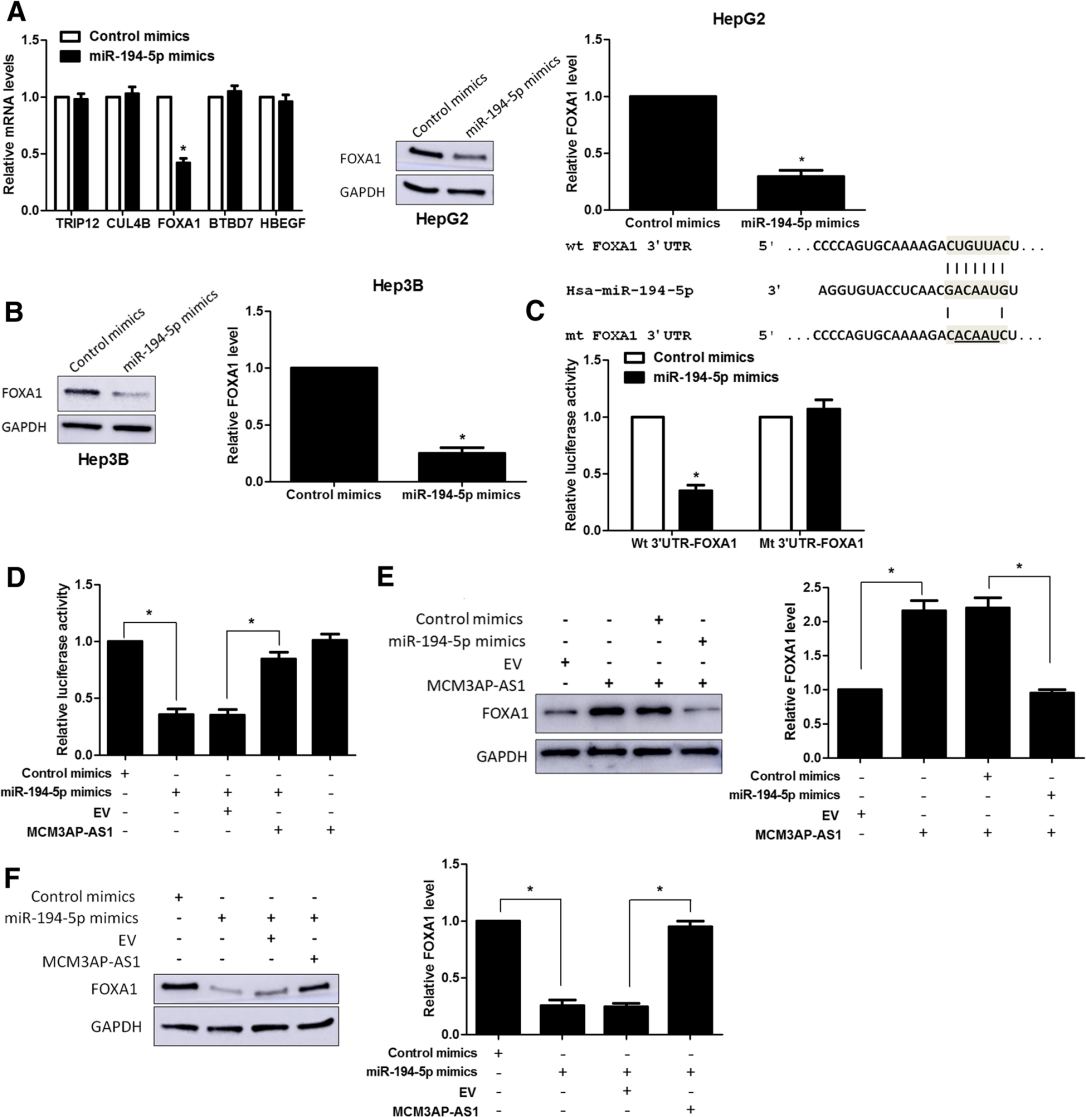
FOXA1 is a direct target of miR-194-5p in HCC cells. **a** HepG2 cells that were transfected with miR-194-5p mimics or control mimics were subjected to qRT-PCR for mRNAs expression. miR-194-5p overexpression reduced the level of FOXA1 protein. **b** miR-194-5p restoration decreased the expression of FOXA1 protein in Hep3B cells. **c** Complementary sequence between miR-194-5p and wild type (wt) 3’UTR of FOXA1. The putative binding sites of miR-194-5p was mutated in 3’UTR of FOXA1 (mt FOXA1 3’UTR). HepG2 cells that were co-transfected with miR-194-5p mimics and wt or mt 3’UTR of FOXA1 were subjected to luciferase activity measurement. **d** HepG2 cells that were co-transfected with MCM3AP-AS1, miR-194-5p mimics and 3’UTR of FOXA1 were subjected to luciferase activity measurement. **e** MCM3AP-AS1 overexpression increased the expression of FOXA1, which was reduced by miR-194-5p restoration in SMMC-7721 cells. **f** miR-194-5p overexpression reduced the level of FOXA1, and this was reversed by MCM3AP-AS1 overexpression in HepG2 cells. *P < 0.05 by Student’s t-test

By using starBase V3.0, we found 48 candidate targets containing the complementary site for the seed region of miR-194-5p in four different prediction databases (targetScan, picTar, PITA and miRanada). Among these genes, only thyroid hormone receptor interactor 12 (TRIP12) [33], cullin 4B (CUL4B) [34], FOXA1 [27, 35, 36], BTB domain containing 7 (BTBD7) [37] and heparin binding EGF like growth factor (HBEGF) [38] were reported to be oncogenes in HCC. Then, we screened the mRNA levels of these genes after miR-194-5p overexpression in HepG2 cells. We found that miR-194-5p overexpression only reduced the level of FOXA1 mRNA (*P* < 0.05, Fig. 6a). Furthermore, FOXA1 protein expression was significantly down-regulated by miR-194-5p in both HepG2 and Hep3B cells (*P* < 0.05, Fig. 6a and b). The expression of FOXA1 protein in tumor tissues from MCM3AP-AS1 knockdown injected mice was significantly lower than that in control mice (*P* < 0.05, Additional file 5: Figure S5B). Furthermore, HCC tissues with high MCM3AP-AS1 or low miR-194-5p level showed an obvious higher level of FOXA1 protein compared to cases with low MCM3AP-AS1 or high miR-194-5p level (*P* < 0.05, Additional file 6: Figure S6D and S6E). Luciferase reporter assay indicated that miR-194-5p overexpression markedly decreased the luciferase activity of vectors containing wt 3’UTR of FOXA1 rather than mt 3’UTR of FOXA1 (*P* < 0.05, Fig. 6c). Moreover, MCM3AP-AS1 abolished the inhibitory effect of miR-194-5p on the luciferase activity of vectors containing wt 3’UTR of FOXA1 (*P* < 0.05, Fig. 6d). Meanwhile, MCM3AP-AS1 overexpression prominently increased FOXA1 expression, which was reversed by miR-194-5p restoration in SMMC-7721 cells (*P* < 0.05, Fig. 6e). Overexpression of miR-194-5p obviously reduced FOXA1 level in HepG2 cells, while FOXA1 downregulation was abrogated by MCM3AP-AS1 overexpression (P < 0.05, Fig. 6f). Taken together, MCM3AP-AS1 functioned as a ceRNA to promote FOXA1 expression by sponging miR-194-5p in HCC.

### FOXA1 restoration attenuates the effect of MCM3AP-AS1 knockdown on HCC cells

**Fig. 7:**
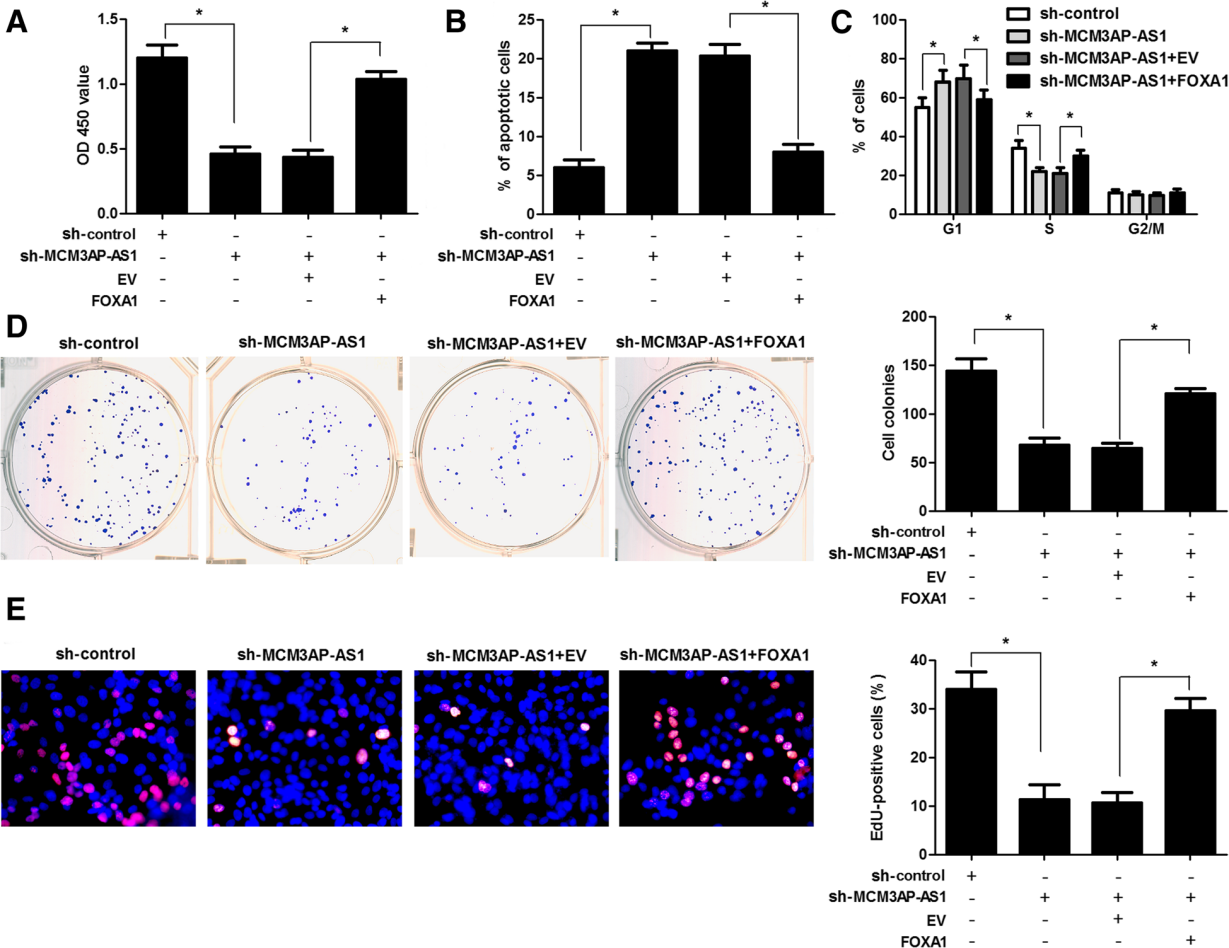
FOXA1 restoration attenuates the role of MCM3AP-AS1 knockdown in HCC cells. HepG2 cells with MCM3AP-AS1 knockdown were transfected with FOXA1 vector or empty vector (EV). **a** CCK-8, (**b)** apoptosis assay, (**c**) cell cycle assay, (**d**) colony formation, and (**e**) EdU incorporation assay were performed to measure cell proliferation, apoptosis and cell cycle progression. *P < 0.05 by Student’s t-test

To explore whether the FOXA1 was critical for cell proliferation restriction, G1 arrest and apoptosis upon MCM3AP-AS1 knockdown, HepG2 cells with MCM3AP-AS1 knockdown were transfected with FOXA1 expression vectors. CCK8 assays indicated that restoration of FOXA1 attenuated the proliferation suppressive role of MCM3AP-AS1 knockdown in HepG2 cells (*P* < 0.05, Fig. 7a). Flow cytometry assays revealed that FOXA1 restoration reversed apoptosis and G1 arrest in HepG2 cells with MCM3AP-AS1 knockdown (P < 0.05, Fig. 7b and c). Moreover, colony formation and EdU incorporation assays also indicated that overexpression of FOXA1 attenuated the growth arrest of HepG2 cells induced by MCM3AP-AS1 knockdown (P < 0.05, Fig. 7d and e). Thus, these results strongly suggested that MCM3PA-AS1 knockdown induced cell proliferation restriction, cell cycle arrest and apoptosis was at least partially mediated by FOXA1 inhibition in HCC.

## Discussion

Tens of thousands lncRNAs are identified by human transcriptome sequencing. Increasing studies have revealed more and more cancer-related lnRNAs, some of them play essential roles in tumorigenesis and progression of HCC [39]. Beside the well characterized lncRNAs, it is still worth to investigate potential essential lncRNAs in controlling HCC initiation and progression. Thus, we re-analyzed the microarray data from public available database about the differentially expressed lncRNAs in HCC. Interestingly, we found a novel lncRNA MCM3AP-AS1, which was markedly overexpressed in HCC tissues and cell lines compared with tumor-adjacent tissues and normal hepatic cell line, respectively. High expression of MCM3AP-AS1 was positively associated with large tumor size, high tumor grade and advanced tumor stage. Moreover, elevated expression of MCM3AP-AS1 indicated poor clinical outcomes of HCC patients. Therefore, our study identified a novel HCC-related lncRNA MCM3AP-AS1, which predicted poor prognosis of HCC patents. A recent study also reports that MCM3AP-AS1 functions as an oncogenic lncRNA and it is overexpressed in glioma-associated endothelial cells (GECs) [40]. TCGA data from OncoLnc also reveals that high expression of MCM3AP-AS1 indicates poor survival of patients with colon adenocarcinoma. Thus, the aberrant expression and clinical significance of MCM3AP-AS1 in other human cancers are worth to be investigated.

Next, loss-of-function assays indicated that MCM3AP-AS1 knockdown inhibited cell proliferation, colony formation and cell cycle progression, and induced apoptosis of HCC cells in vitro. In vivo experiments found that MCM3AP-AS1 silencing suppressed HCC tumor growth in mice. Taken together, these results suggested an oncogenic role of MCM3AP-AS1 in HCC. MCM3AP-AS1 promotes angiogenesis of glioblastoma in vitro [40]. This implies that MCM3AP-AS1 may regulate other malignant behaviors of HCC cells including metastasis and angiogenesis, which needs further study.

One of the most popular functional model for lncRNAs is that lncRNAs function as ceRNAs to sponge miRNAs via sequence complementarity and subsequently influence functional roles of miRNAs [39]. Yang et al. find that MCM3AP-AS1 acts as a ceRNA to promote KLF5/AGGF1 axis, and activate PI3K/AKT and ERK1/2 signaling pathways by sponging miR-211 in glioblastoma [40]. Here, we found that MCM3AP-AS1 mainly located in the cytoplasm and the abundance of MCM3AP-AS1 was comparable to that of miR-194-5p in HCC cells. Moreover, MCM3AP-AS1 silencing prominently up-regulated miR-194-5p expression in HCC cells. An inverse correlation between MCM3AP-AS1 and miR-194-5p expression was confirmed in HCC tissues from our cohort and TCGA database. The following luciferase reporter assay and RNA pull down assay demonstrated that MCM3AP-AS1 acted as molecular sponge for miR-194-5p by directly binding to complementary sequence in HCC cells. Since miR-194-5p suppresses cell proliferation and blocks G1-S transition in HCC cells [41]. And a recent study also reports the lncRNA XIST regulation of miR-194-5p in HCC [42]. Thus, we considered that MCM3AP-AS1 played an oncogenic role in HCC via down-regulating miR-194-5p expression. FOXA1, a transcription factor, promotes tumor growth of HCC [27, 35, 36]. Our previous study shows that FOXA1 mediates the tumor suppressive role of miR-212 in HCC tumor growth [27]. MSL2 contributes to the growth of HCC cells in vitro and in vivo and is up-regulated HBx-mediated activation of YAP/FOXA1 signaling [35]. Moreover, tumor suppressive lncRNA MT1DP inhibits cell proliferation and colony formation, but induces apoptosis of HCC cell in a FOXA1 dependent manner [36]. In this study, FOXA1 was identified as a direct target of miR-194-5p in HCC cells. miR-194-5p regulation of FOXA1 is also observed in lung cancer [43]. Notably, MCM3AP-AS1 positively regulated FOXA1 abundance in HCC cells, while miR-194-5p showed an opposite regulatory effect. A positive correlation between MCM3AP-AS1 and FOXA1 and a negative correlation between miR-194-5p and FOXA1 were observed in HCC tissues. Importantly, FOXA1 restoration reversed MCM3AP-AS1 knockdown induced HCC cell proliferation restriction, cell cycle arrest and apoptosis. To conclude, our study provided a novel insight that MCM3AP-AS1/miR-194-5p/FOXA1 axis contributed to the growth of HCC. MCM3AP-AS1/miR-194-5p/FOXA1 axis might be potential therapeutic targets for HCC.

## Conclusions

In summary, our findings identified a novel lncRNA MCM3AP-AS1, which was up-regulated in HCC and associated with poor prognosis of HCC patients. MCM3AP-AS1 knockdown inhibited the proliferation, cell cycle progression and induced apoptosis of HCC cells, and suppressed tumor growth of HCC in vivo. Mechanistically, MCM3AP-AS1 functioned as an oncogenic lncRNA by acting as a ceRNA to sponge miR-194-5p and subsequently promoted FOXA1 expression. Our data suggested that MCM3AP-AS1 might be a potential prognostic biomarker and therapeutic target for HCC.

## Additional files

Additional file 1: **Figure S1.** The expression of MCM3AP-AS1 between HCC and normal liver tissues in TCGA database. The levels of MCM3AP-AS1 in HCC tissues were obviously higher than that in normal liver tissues in TCGA database from starBase V3.0 platform. *P* < 0.0001 by Student’s t-test. (TIF 198 kb)

Additional file 2: **Figure S2.** MCM3AP-AS1 knockdown induced apoptosis of HCC cells. MCM3AP-AS1 knockdown increased the levels of cleaved PARP1, cleaved caspase-3, cleaved caspase-7 and p21, and reduced the expression of Cyclin D1 in HepG2 and Hep3B cells. *P < 0.05 by Student’s t-test versus sh-control. (TIF 304 kb)

Additional file 3: **Figure S3.** MCM3AP-AS1 knockdown did not impact the growth of LO2 cells. LO2 cells were transfected with MCM3AP-AS1 shRNAs and control shRNA. (A) CCK-8, (B) colony formation, (C) EdU incorporation assay, (D) apoptosis assay, and (E) cell cycle assay were performed to measure cell proliferation, apoptosis and cell cycle progression. (TIF 104 kb)

Additional file 4: **Figure S4.** The prognostic significance of miR-194-5p and miR-23c in HCC based on TCGA database. Kaplan-Meier survival analysis revealed that HCC patients with low miR-194-5p (miR-23c) expression showed a significant poorer overall survival compared to those with high miR-194-5p (miR-23c) expression based on TCGA data from OncoLnc platform. *P* = 0.00808 and 0.00319 by Log-rank test. (TIF 137 kb)

Additional file 5: **Figure S5.** The expression of miR-194-5p and FOXA1 in xenograft tissues. Xenograft tissues arising from MCM3AP-AS1 knockdown group (*n* = 6) and control group (n = 6) were subjected to qRT-PCR and immunoblotting for (A) miR-194-5p and (B) FOXA1 protein expression, respectively. *P < 0.05 by Student’s t-test. (TIF 169 kb)

Additional file 6: **Figure S6.** The relationships among MCM3AP-AS1, miR-194-5p and FOXA1 expression in HCC. (A) The expression of miR-194-5p in HCC tissues (*n* = 80) was markedly lower than that in paracancerous tissues (n = 80). P < 0.0001 by Student’s t-test. (B) A negative correlation between MCM3AP-AS1 and miR-194-5p expression was observed in 80 cases of HCC tissues. r = − 0.2489, *P* = 0.0260 by Spearman correlation test. (C) MCM3AP-AS1 was inversely correlated with miR-194-5p expression in HCC tissues based on TCGA database from starBase V3.0 platform. r = − 0.159, *P* = 0.00222 by Spearman correlation test. (D) Immunoblotting analysis revealed that the expression of FOXA1 protein in HCC tissues with high MCM3AP-AS1 level (*n* = 40) was significantly higher than that in HCC tissues with low MCM3AP-AS1 level (n = 40). *P < 0.05 by Student’s t-test. (E) Immunoblotting analysis revealed that the expression of FOXA1 protein in HCC tissues with low miR-194-5p level (n = 40) was significantly higher than that in HCC tissues with high miR-194-5p level (n = 40). *P < 0.05 by Student’s t-test. (TIF 522 kb)

Additional file 7: **Figure S7.** miR-194-5p inversely regulates MCM3AP-AS1 expression in HCC cells. (A) HepG2 cells that were co-transfected with miR-194-5p mimics and wt or mt MCM3AP-AS1 vector were measured for luciferase activity. (B) miR-194-5p overexpression obviously reduced the expression of MCM3AP-AS1 in HepG2 and Hep3B cells. *P < 0.05 by Student’s t-test. (TIF 54 kb)

Additional file 8 **Figure S8.** The subcellular location and copy number of MCM3AP-AS1 in HCC cells. (A) RNA fluorescent in situ hybridization indicated MCM3AP-AS1 mainly located in cytoplasm of Hep3B and HepG2 cells. (B) qRT-PCR was performed to measure the copy numbers of MCM3AP-AS1 and miR-194-5p in HepG2 and Hep3B cells. (TIF 679 kb)

## Abbreviations

ANXA2: Annexin A2
BTBD7: BTB domain containing 7
ceRNA: Competing endogenous RNA
CTNNBIP1: Catenin beta interacting protein 1
CUL4B: Cullin 4B
EMT: Epithelial-mesenchymal transition
EphA4: Eph tyrosine kinase receptor A4
FBXW7: F-box and WD repeat domain containing 7
FOXA2: Forkhead box A2
GECs: Glioma-associated endothelial cells
GEO: Gene Expression Omnibus
HBEGF: Heparin binding EGF like growth factor
HCC: Hepatocellular carcinoma
HNRNPA2B1: Heterogeneous nuclear ribonucleoprotein A2/B1
MCM3AP-AS1: MCM3AP antisense RNA 1
NCBI: National Center for Biotechnology Information
ncRNAs: Non-coding RNAs
NF-κB: Nuclear factor κB
RFA: Radiofrequency ablation
TACE: Transcatheter arterial chemoembolization
TRIP12: Thyroid hormone receptor interactor 12
