# Supplementary figures and images for: A novel lncRNA MCM3AP-AS1 promotes the growth of hepatocellular carcinoma by targeting miR-194-5p/FOXA1 axis
Source: Mol Cancer. 2019 Feb 19;18:28. doi: 10.1186/s12943-019-0957-7 (PMC6381672; doi:10.1186/s12943-019-0957-7)

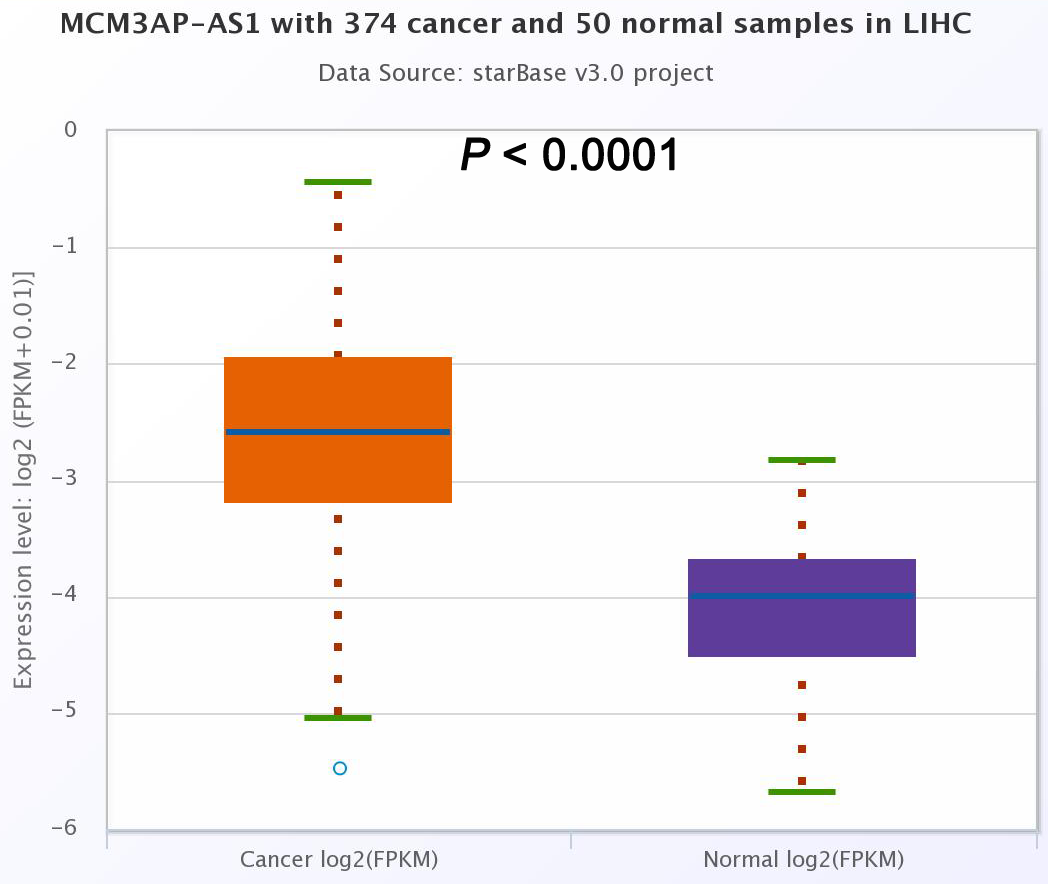

Supplement: Supplementary file 1 — Figure S1. The expression of MCM3AP-AS1 between HCC and normal liver tissues in TCGA database. The levels of MCM3AP-AS1 in HCC tissues were obviously higher than that in normal liver tissues in TCGA database from starBase V3.0 platform. P < 0.0001 by Student’s t-test. (TIF 198 kb) [file 12943_2019_957_MOESM1_ESM.tif]

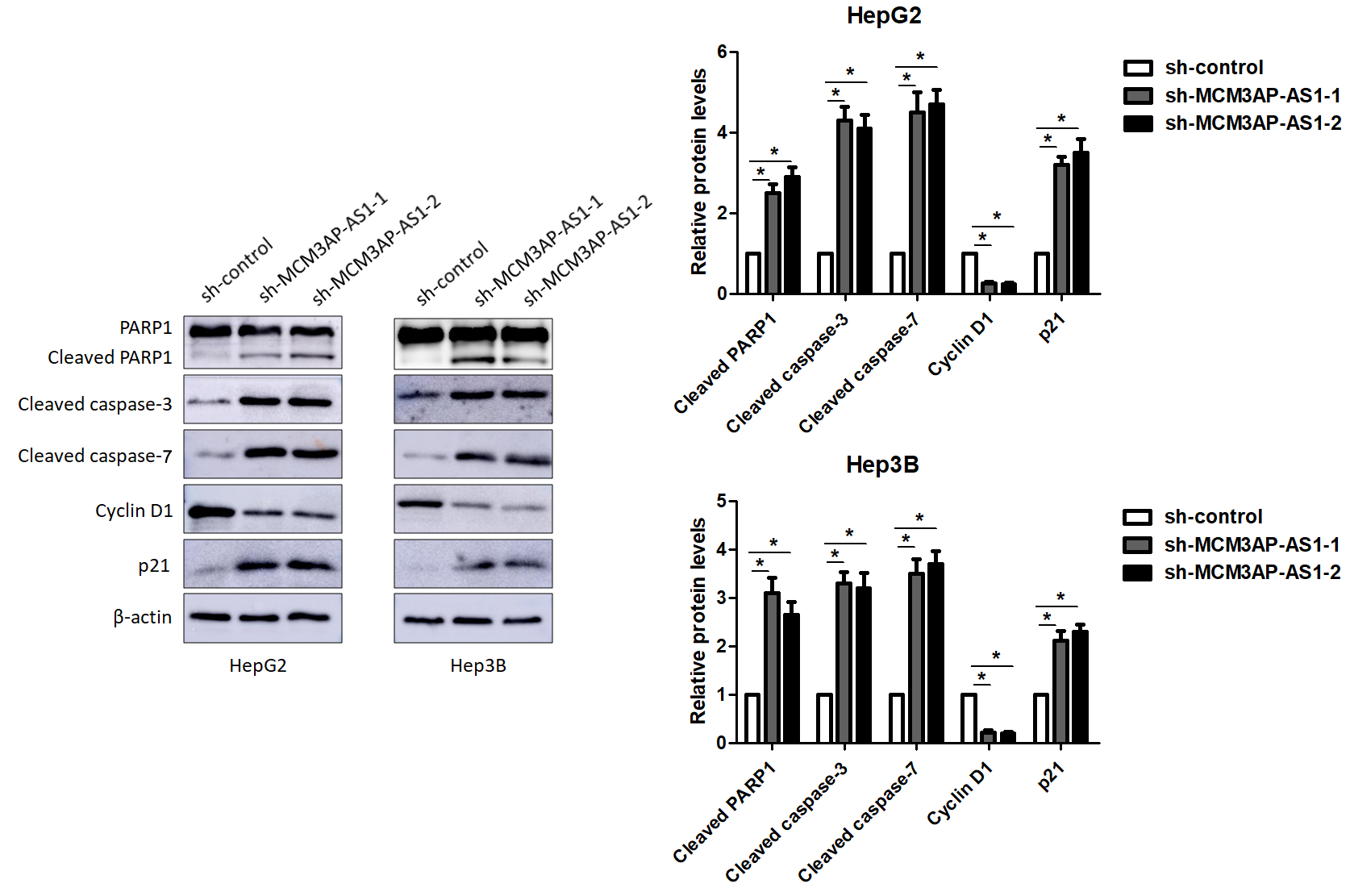

Supplement: Supplementary file 2 — Figure S2. MCM3AP-AS1 knockdown induced apoptosis of HCC cells. MCM3AP-AS1 knockdown increased the levels of cleaved PARP1, cleaved caspase-3, cleaved caspase-7 and p21, and reduced the expression of Cyclin D1 in HepG2 and Hep3B cells. *P < 0.05 by Student’s t-test versus sh-control. (TIF 304 kb) [file 12943_2019_957_MOESM2_ESM.tif]

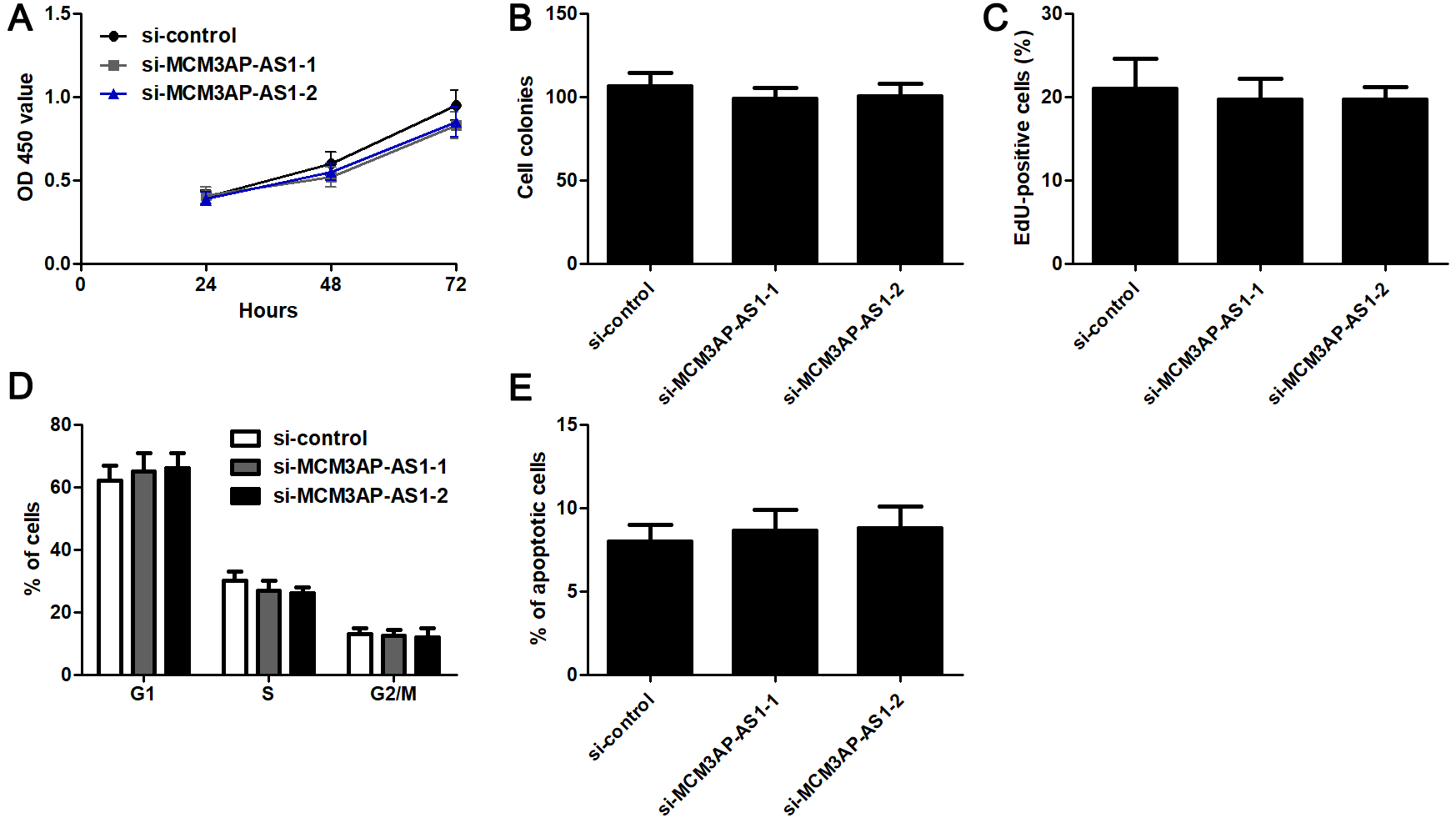

Supplement: Supplementary file 3 — Figure S3. MCM3AP-AS1 knockdown did not impact the growth of LO2 cells. LO2 cells were transfected with MCM3AP-AS1 shRNAs and control shRNA. (A) CCK-8, (B) colony formation, (C) EdU incorporation assay, (D) apoptosis assay, and (E) cell cycle assay were performed to measure cell proliferation, apoptosis and cell cycle progression. (TIF 104 kb) [file 12943_2019_957_MOESM3_ESM.tif]

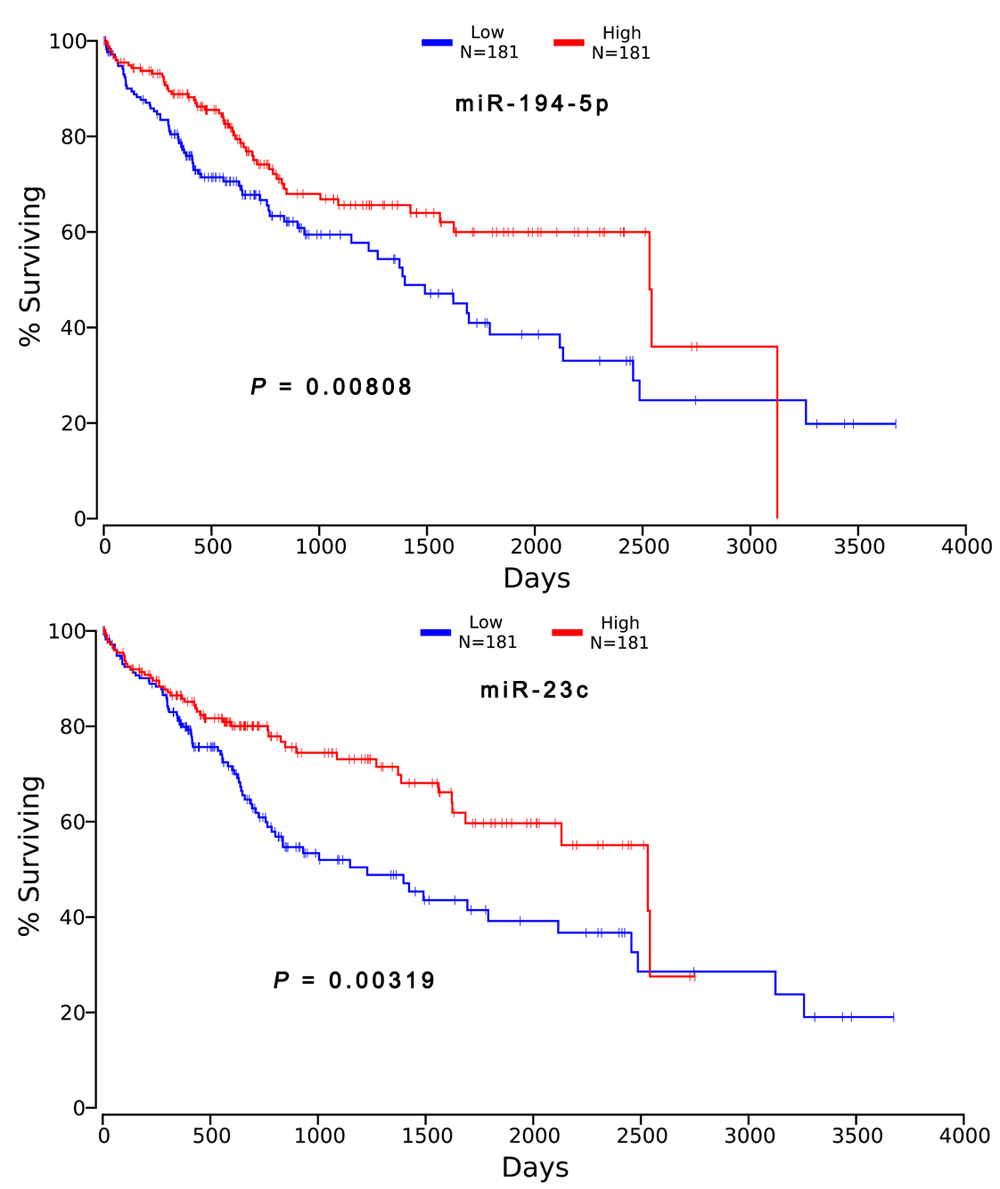

Supplement: Supplementary file 4 — Figure S4. The prognostic significance of miR-194-5p and miR-23c in HCC based on TCGA database. Kaplan-Meier survival analysis revealed that HCC patients with low miR-194-5p (miR-23c) expression showed a significant poorer overall survival compared to those with high miR-194-5p (miR-23c) expression based on TCGA data from OncoLnc platform. P = 0.00808 and 0.00319 by Log-rank test. (TIF 137 kb) [file 12943_2019_957_MOESM4_ESM.tif]

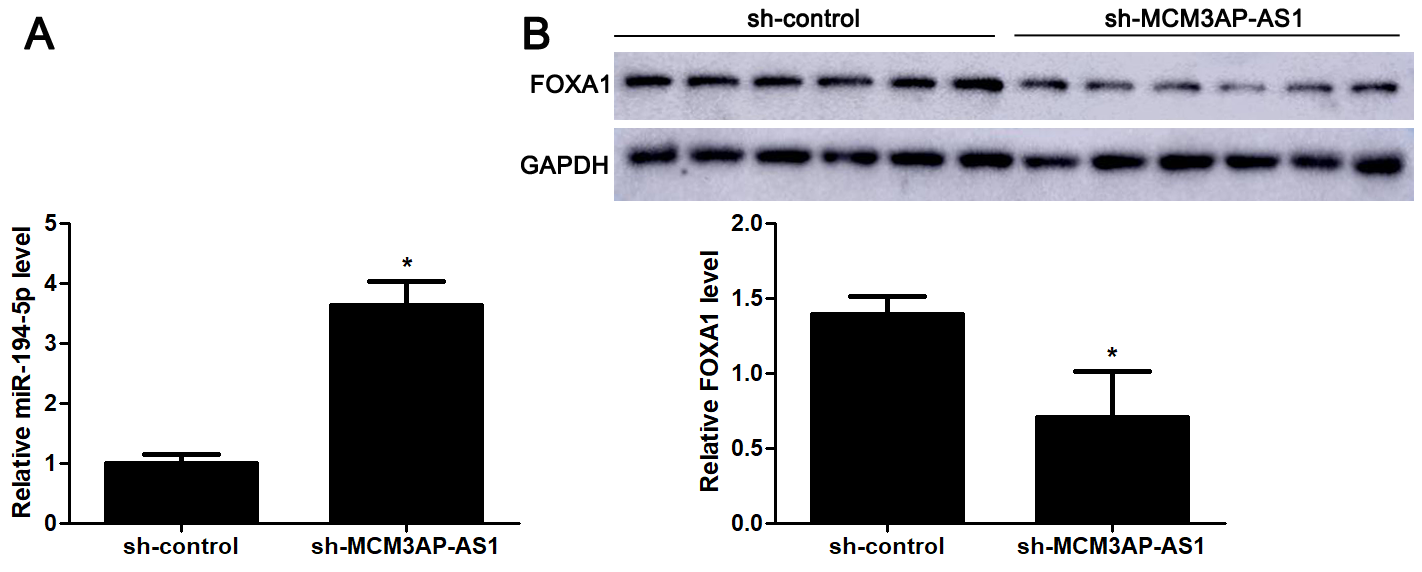

Supplement: Supplementary file 5 — Figure S5. The expression of miR-194-5p and FOXA1 in xenograft tissues. Xenograft tissues arising from MCM3AP-AS1 knockdown group (n = 6) and control group (n = 6) were subjected to qRT-PCR and immunoblotting for (A) miR-194-5p and (B) FOXA1 protein expression, respectively. *P < 0.05 by Student’s t-test. (TIF 169 kb) [file 12943_2019_957_MOESM5_ESM.tif]

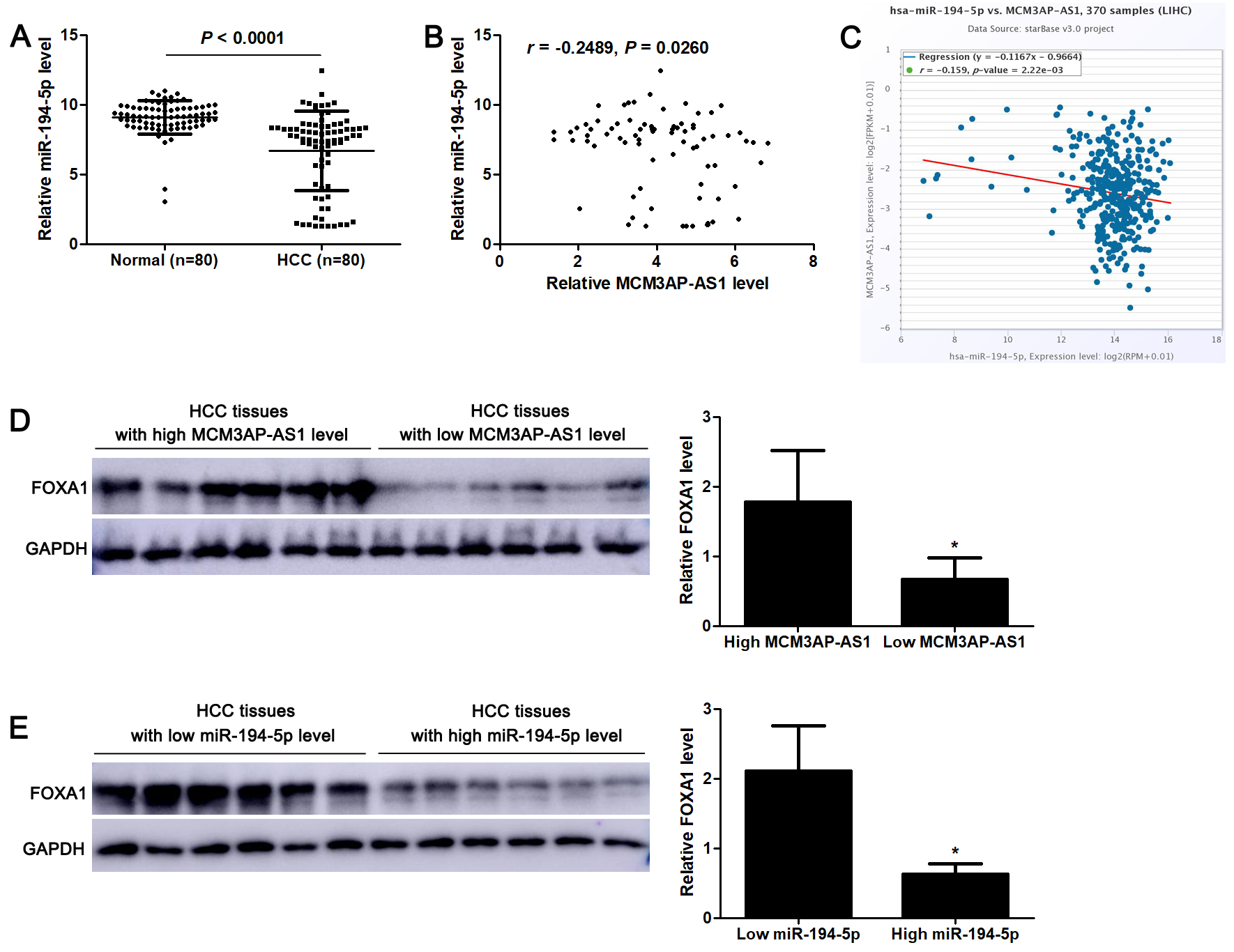

Supplement: Supplementary file 6 — Figure S6. The relationships among MCM3AP-AS1, miR-194-5p and FOXA1 expression in HCC. (A) The expression of miR-194-5p in HCC tissues (n = 80) was markedly lower than that in paracancerous tissues (n = 80). P < 0.0001 by Student’s t-test. (B) A negative correlation between MCM3AP-AS1 and miR-194-5p expression was observed in 80 cases of HCC tissues. r = − 0.2489, P = 0.0260 by Spearman correlation test. (C) MCM3AP-AS1 was inversely correlated with miR-194-5p expression in HCC tissues based on TCGA database from starBase V3.0 platform. r = − 0.159, P = 0.00222 by Spearman correlation test. (D) Immunoblotting analysis revealed that the expression of FOXA1 protein in HCC tissues with high MCM3AP-AS1 level (n = 40) was significantly higher than that in HCC tissues with low MCM3AP-AS1 level (n = 40). *P < 0.05 by Student’s t-test. (E) Immunoblotting analysis revealed that the expression of FOXA1 protein in HCC tissues with low miR-194-5p level (n = 40) was significantly higher than that in HCC tissues with high miR-194-5p level (n = 40). *P < 0.05 by Student’s t-test. (TIF 522 kb) [file 12943_2019_957_MOESM6_ESM.tif]

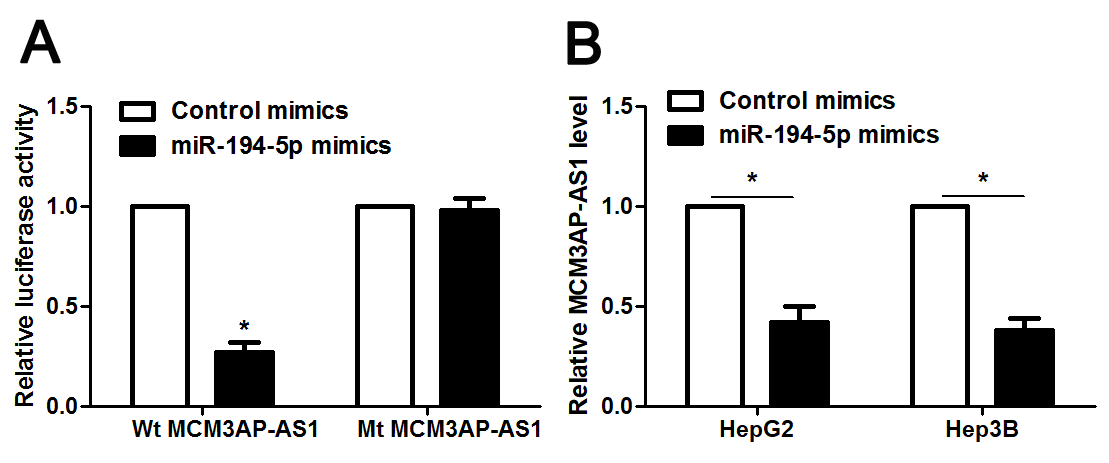

Supplement: Supplementary file 7 — Figure S7. miR-194-5p inversely regulates MCM3AP-AS1 expression in HCC cells. (A) HepG2 cells that were co-transfected with miR-194-5p mimics and wt or mt MCM3AP-AS1 vector were measured for luciferase activity. (B) miR-194-5p overexpression obviously reduced the expression of MCM3AP-AS1 in HepG2 and Hep3B cells. *P < 0.05 by Student’s t-test. (TIF 54 kb) [file 12943_2019_957_MOESM7_ESM.tif]

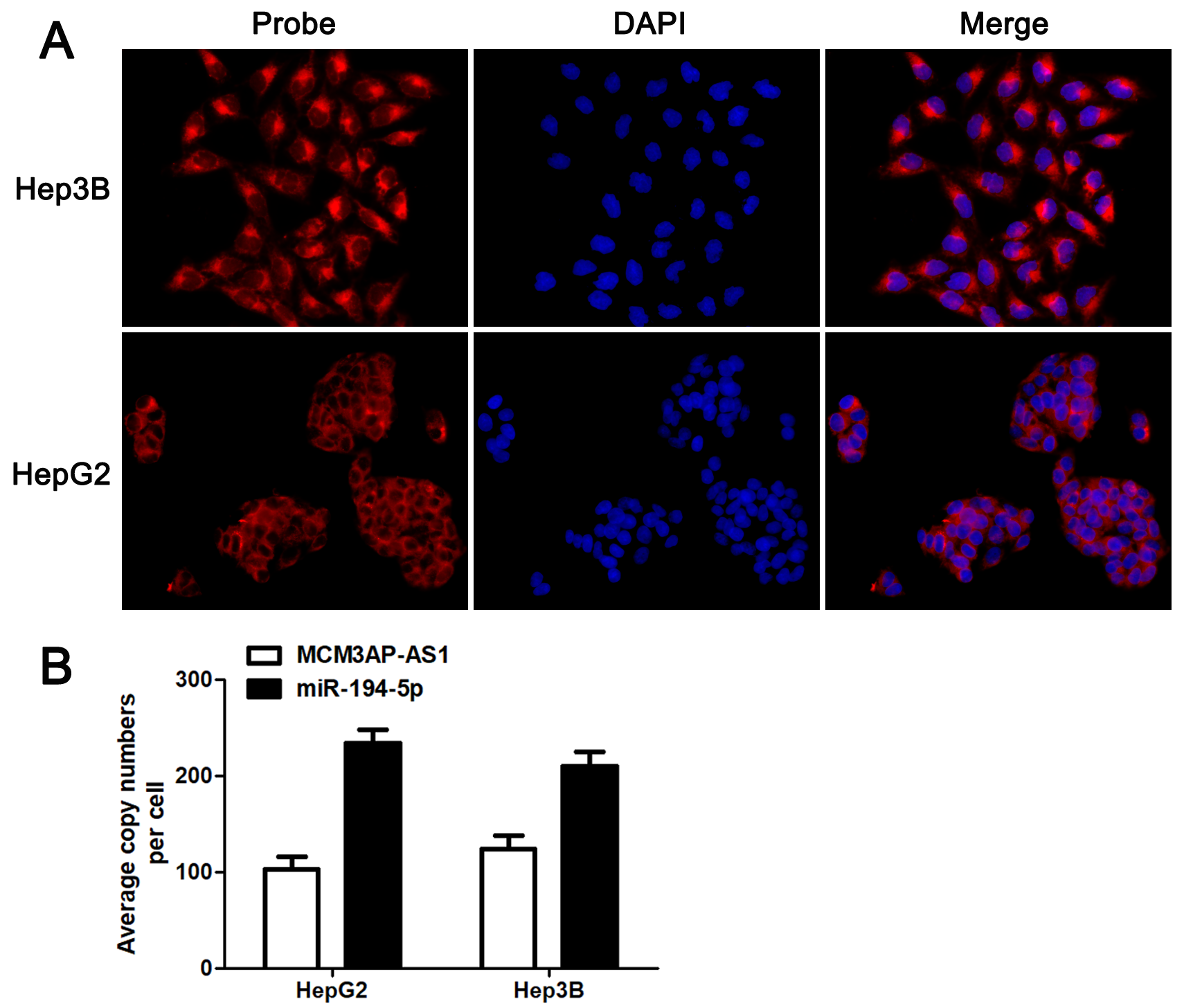

Supplement: Supplementary file 8 — Figure S8. The subcellular location and copy number of MCM3AP-AS1 in HCC cells. (A) RNA fluorescent in situ hybridization indicated MCM3AP-AS1 mainly located in cytoplasm of Hep3B and HepG2 cells. (B) qRT-PCR was performed to measure the copy numbers of MCM3AP-AS1 and miR-194-5p in HepG2 and Hep3B cells. (TIF 679 kb) [file 12943_2019_957_MOESM8_ESM.tif]
